# Human immunoglobulin repertoire analysis guides design of vaccine priming immunogens targeting HIV V2-apex broadly neutralizing antibody precursors

**DOI:** 10.1016/j.immuni.2022.09.001

**Published:** 2022-11-08

**Authors:** Jordan R. Willis, Zachary T. Berndsen, Krystal M. Ma, Jon M. Steichen, Torben Schiffner, Elise Landais, Alessia Liguori, Oleksandr Kalyuzhniy, Joel D. Allen, Sabyasachi Baboo, Oluwarotimi Omorodion, Jolene K. Diedrich, Xiaozhen Hu, Erik Georgeson, Nicole Phelps, Saman Eskandarzadeh, Bettina Groschel, Michael Kubitz, Yumiko Adachi, Tina-Marie Mullin, Nushin B. Alavi, Samantha Falcone, Sunny Himansu, Andrea Carfi, Ian A. Wilson, John R. Yates, James C. Paulson, Max Crispin, Andrew B. Ward, William R. Schief

**Affiliations:** 1 Center for HIV/AIDS Vaccine Development, The Scripps Research Institute, La Jolla, CA 92037, USA; 2 IAVI Neutralizing Antibody Center, The Scripps Research Institute, La Jolla, CA 92037, USA; 3 Department of Immunology and Microbial Science, The Scripps Research Institute, La Jolla, CA 92037, USA; 4 Department of Integrative Structural and Computational Biology, The Scripps Research Institute, La Jolla, CA 92037, USA; 5 School of Biological Sciences, University of Southampton, Southampton SO17 1BJ, UK; 6 Department of Molecular Medicine, The Scripps Research Institute, La Jolla, CA 92037, USA; 7 Moderna Inc., Cambridge, MA 02139, USA; 8 Ragon Institute of MGH, MIT, and Harvard, Cambridge, MA 02139, USA

**Keywords:** AIDS vaccines, structural vaccinology, HIV antibodies, immunoinformatics, germline targeting

## Abstract

Broadly neutralizing antibodies (bnAbs) to the HIV envelope (Env) V2-apex region are important leads for HIV vaccine design. Most V2-apex bnAbs engage Env with an uncommonly long heavy-chain complementarity-determining region 3 (HCDR3), suggesting that the rarity of bnAb precursors poses a challenge for vaccine priming. We created precursor sequence definitions for V2-apex HCDR3-dependent bnAbs and searched for related precursors in human antibody heavy-chain ultradeep sequencing data from 14 HIV-unexposed donors. We found potential precursors in a majority of donors for only two long-HCDR3 V2-apex bnAbs, PCT64 and PG9, identifying these bnAbs as priority vaccine targets. We then engineered ApexGT Env trimers that bound inferred germlines for PCT64 and PG9 and had higher affinities for bnAbs, determined cryo-EM structures of ApexGT trimers complexed with inferred-germline and bnAb forms of PCT64 and PG9, and developed an mRNA-encoded cell-surface ApexGT trimer. These methods and immunogens have promise to assist HIV vaccine development.

## Highlights

• Immunoinformatics reveal precursors for V2-apex bnAbs PCT64 and PG9 in HIV-negative donors
• Immunogen design produces ApexGT trimers with affinity for inferred precursors
• Cryo-EM structures of trimers bound to precursors or bnAbs assist immunogen design
• Membrane-anchored ApexGT trimers retain antigenicity on DNA- or mRNA-transfected cells

## Graphical abstract

**Figure:**
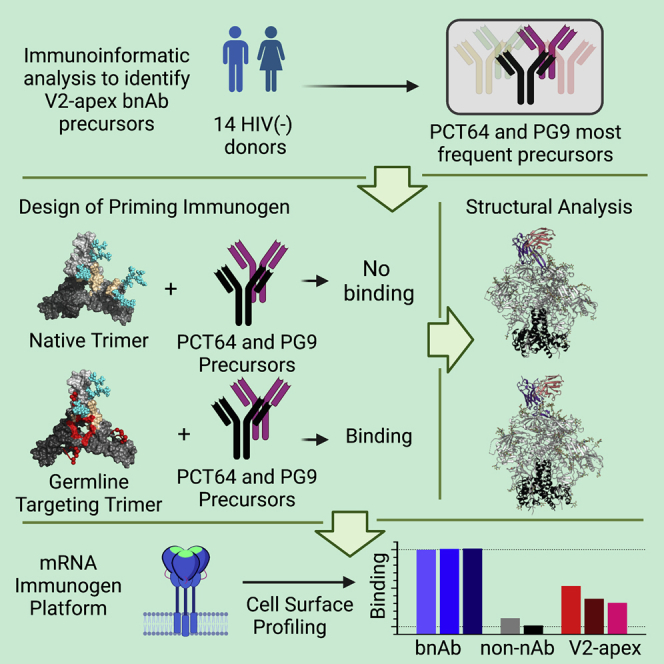

## Teaser

Broadly neutralizing antibodies (bnAbs) to the HIV envelope V2-apex are important leads for vaccine design but pose major challenges for precursor priming due to long heavy-chain complementarity-determining region 3 (HCDR3). We prioritize two V2-apex bnAbs, PCT64 and PG9, design and characterize ApexGT trimers that bind bnAb precursors, and develop membrane-bound trimers for mRNA delivery as candidate priming immunogens.

## Introduction

A vaccine for HIV-1 is urgently needed, as there are approximately 1.5 million new infections each year as of 2020 (http://www.unaids.org/en/resources/fact-sheet). The target of HIV neutralizing antibodies, the trimeric envelope (Env) spike, varies substantially in sequence across different HIV-1 isolates, indicating that a vaccine should induce “broadly neutralizing antibodies” (bnAbs), antibodies capable of neutralizing diverse isolates (Burton and Hangartner, 2016). Potent HIV bnAbs develop in a small percentage of infected individuals, typically over an extended course of infection (Burton and Mascola, 2015; Kwong and Mascola, 2018). Passive immunization with HIV-1 bnAbs has been shown to protect against simian/human immunodeficiency virus challenge in non-human primates (Pegu et al., 2017, 2019) and be capable of protecting humans against HIV-1 infection by neutralization-sensitive isolates (Corey et al., 2021). Vaccine induction of bnAbs is regarded as having potential to protect against HIV, but bnAb elicitation in humans has not yet been achieved.

HIV bnAbs target at least five major epitopic regions on the Env trimer: V2-apex, V3-glycan, CD4 binding site, gp120/gp41 interface, and membrane proximal external region (MPER). Here, we focus on V2-apex bnAbs. These have been isolated from multiple individuals and include some of the most potent bnAbs. V2-apex-directed responses are present in the serum of 15%–20% of individuals who produce bnAbs (Landais et al., 2016; Walker et al., 2010); thus the human immune system appears to be relatively well-suited to generate responses to this epitope region.

Seven classes of V2-apex bnAbs have been identified, five of which possess long, negatively charged HCDR3s that are often decorated with sulfated tyrosines: PG9/PG16 (Walker et al., 2009), PGT141-145 and PGDM1400-1412 (Sok et al., 2014; Walker et al., 2011), CH01-CH04 (Bonsignori et al., 2011), the CAP256.VRC26 lineage (Doria-Rose et al., 2014, 2016), and the PCT64 lineage (Landais et al., 2017). HCDR3s for these bnAbs mediate binding by reaching through the conserved glycan shield to contact a positively charged, semi-conserved protein surface on strand C of the V2 loop (Andrabi et al., 2015, 2017; Gorman et al., 2016; Landais et al., 2017; Lee et al., 2017; McLellan et al., 2011; Pancera et al., 2010, 2013; Pejchal et al., 2010; Sok et al., 2014). Vaccine elicitation of V2-apex bnAbs may therefore require eliciting antibodies with HCDR3s similar to sequences in the known bnAbs.

The success of any vaccine strategy to induce V2-apex bnAbs with HCDR3s similar to those in the known bnAbs will depend strongly on whether the priming immunogen can activate human naive B cells with appropriate germline-recombined HCDR3s that have potential to mature into bnAb HCDR3s (Steichen et al., 2019). The ability of a priming immunogen to activate appropriate precursors will also depend on the frequency of such precursors in the naive human B cell repertoire—the lower the frequency, the more difficult priming will be. Precursor frequency is a major concern for V2-apex bnAb precursor priming because the very long HCDR3s of many V2-apex bnAbs suggest that precursor frequencies may be extremely low (Briney et al., 2012). Multiple candidates have been proposed or investigated as priming immunogens for V2-apex bnAb responses (Alam et al., 2013; Andrabi et al., 2019; Bhiman et al., 2015; Bonsignori et al., 2011; Doria-Rose et al., 2014; Gorman et al., 2016; Medina-Ramirez et al., 2017), and at least one such candidate has entered clinical trials (BG505 SOSIP.GT1.1 gp140; ClinicalTrials.gov Identifier: NCT04224701). However, none of the candidates have been shown to prime bnAb precursors using B cell receptor (BCR) sequencing of induced responses in an animal model with low precursor frequency approximating the human physiological range, as has been accomplished with germline-targeting immunogens for other bnAb classes (Huang et al., 2020; Jardine et al., 2015; Parks et al., 2019; Sok et al., 2016; Steichen et al., 2019; Tian et al., 2016; Wang et al., 2021).

In this study, we used structural modeling, analysis of VDJ recombination of known V2-apex bnAbs, and bioinformatic analysis of ultradeep next-generation sequencing (NGS) of human BCR heavy chains (HCs) from 14 HIV-unexposed donors to assess the relative frequencies of potential precursors. Our analysis revealed major differences in potential precursor frequencies, with two classes, PCT64 and PG9, having the highest and most consistent frequencies among donors. Potential precursors for these two bnAb classes, whether inferred-germline (iGL) or least-mutated common ancestor (LMCA) precursors, had no detectable binding to native-like trimers from HIV isolate BG505. We then engineered BG505-based trimer immunogens (ApexGT immunogens) that bind to inferred germlines for PCT64 and PG9, and we characterized these immunogens by cryo-electron microscopy (cryo-EM). Finally, we developed mRNA-encoded membrane-bound ApexGT trimer vaccine candidates. In a companion manuscript, immunogenicity studies are reported for protein and mRNA-encoded ApexGT immunogens in PCT64 precursor knockin mouse models under conditions of very low precursor frequencies approximating the human physiological range and with BCR sequencing to prove whether or not targeted responses have been primed (Melzi et al., 2022). These results suggest that a germline-targeting strategy may be possible for two classes of HCDR3-dominant V2-apex bnAbs.

## Results

### Immunoinformatic analysis indicates that PCT64 and PG9/16 are the most promising V2-apex bnAb targets for germline-targeting vaccine design

**Figure 1:**
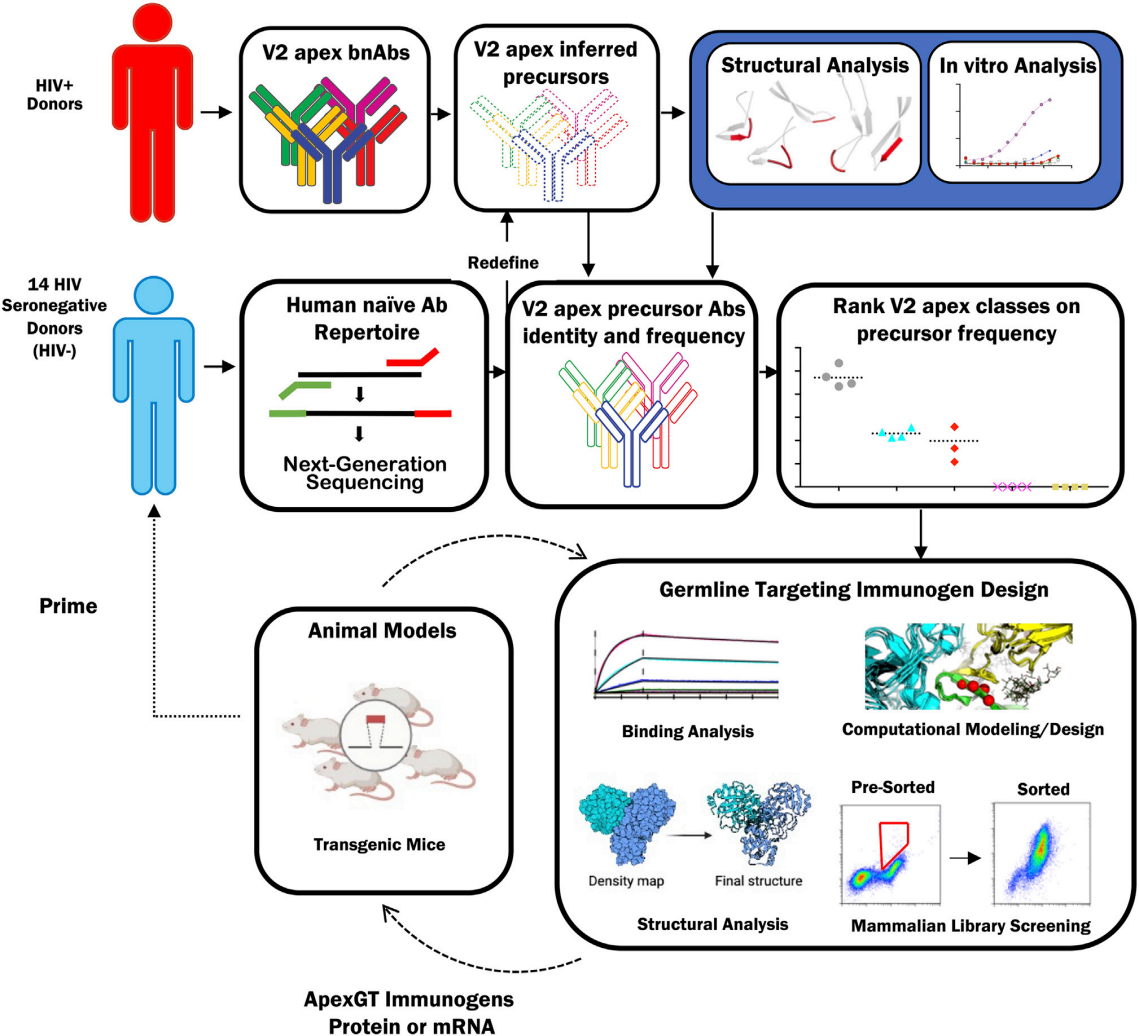
Human antibody repertoire analysis guides V2-apex germline-targeting immunogen design Repertoire analysis, immunogen design, and structure determination (steps with solid arrows) were carried out in this study. Immunization in a knockin mouse model (dashed arrows) was carried out in a companion manuscript (Melzi et al., 2022).

Immunoinformatic analysis of human immunoglobulin repertoires provided guidance for subsequent structure-guided design and testing of V2-apex germline-targeting vaccine-priming immunogens (Figure 1). To begin, we developed precursor definitions for five classes of V2-apex HCDR3-dominant bnAbs. These include the PCT64 lineage (PCT64 donor), CH01-CH04 bnAbs (CH0219 donor), PG9/PG16 bnAbs (IAVI 24 donor), PGT141-145 and PGDM1400-1414 bnAbs (IAVI 84 donor), and the CAP256-VRC26 lineage (CAP256 donor) (Bonsignori et al., 2011; Doria-Rose et al., 2014, 2016; Landais et al., 2017; Walker et al., 2009, 2011). These classes range in neutralization breadth at IC_50_ < 50 μg/mL from 27% (PCT64 35S) to 62% (PG9) and in median IC_50_ potency from 0.62 ug/mL (PCT64 35S) to 0.005 ug/mL (CAP256-VRC26.09) (Table S1). We did not address two V2-apex bnAb classes with Env binding modes not dominated by HCDR3, VRC38, and BG1 (Cale et al., 2017; Freund et al., 2017; Kwong and Mascola, 2018; Wang et al., 2017).

**Figure 2:**
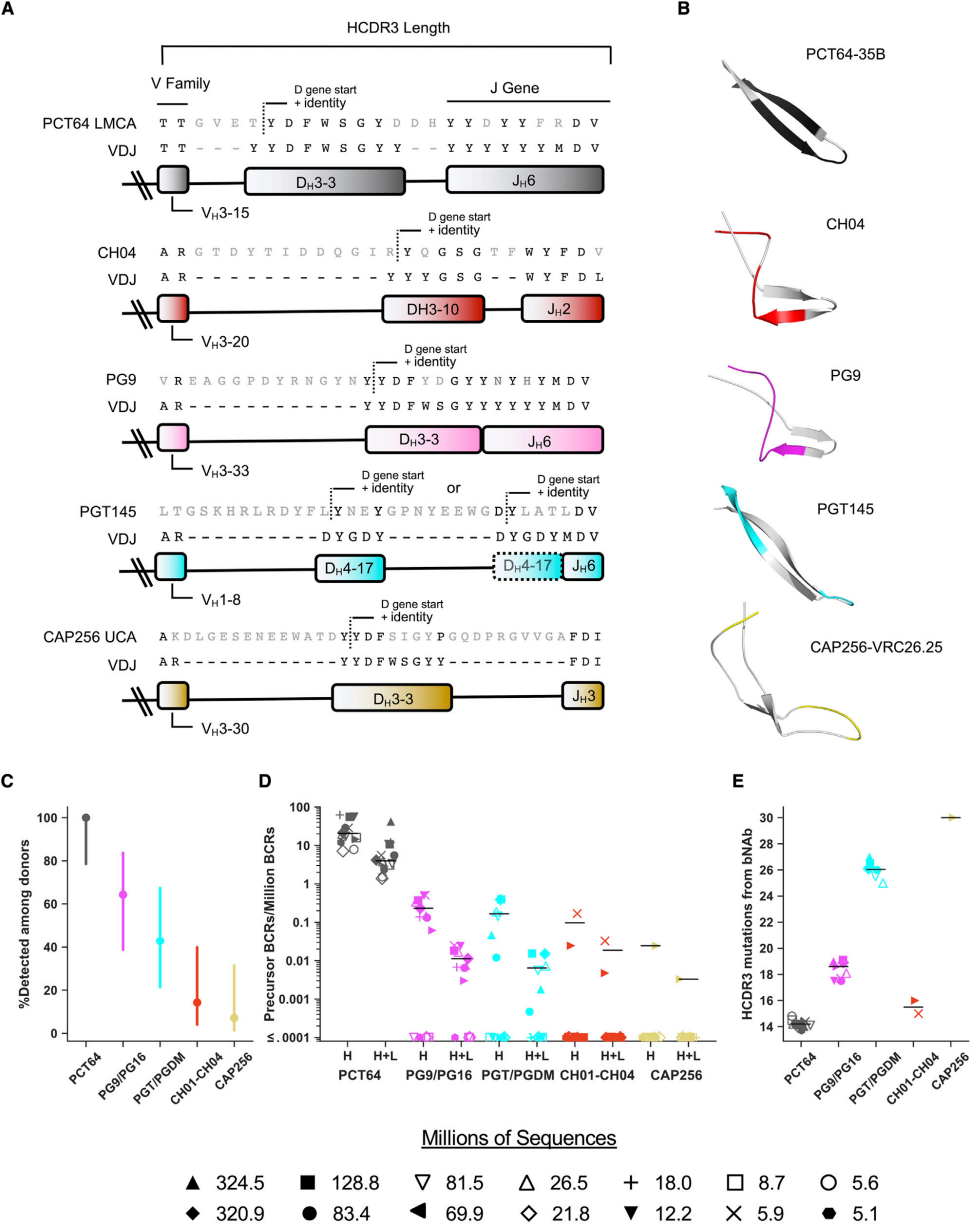
Analysis of precursor frequency and HCDR3 distance to bnAb identifies PCT64 as best and PG9/16 as second-best targets for vaccine design (A) HCDR3 junctions for HCDR3-dominant V2-apex bnAbs aligned with the inferred V_H_, D_H_, and J_H_ gene sequences. Amino acids in gray are either located within a junction (non-templated) or mutated from a germline gene. (B) HCDR3 structures for each class of HCDR3-dominant V2-apex antibody: PCT64-35B (PDB:5FEH), CH04 (PDB:3TCL), PGDM1400 (PDB:4RQQ), PGT145 (PDB:5V8L), and CAP256-VRC26.25 (PDB:5DT1). Templated portions of the HCDR3 are colored as in (A). (C) Precursor detection rates among 14 donors, with 95% confidence intervals computed by the Wilson score method (Agresti and Coull, 1998). Positive detection was defined as at least one precursor being found in a donor. (D) Precursor frequencies for each donor, and for each class of HCDR3-dominant V2-apex bnAb, considering HC only (H) or heavy and light chains (H+L). The number of unique sequences used in the precursor search for each donor is indicated. Black lines indicate median precursor frequencies computed over non-zero values, because the zero values (non-responders) are accounted for in (C). (E) The mean number of mutations to a known bnAb in the class for each precursor found in (D). Each symbol represents the mean for one donor. Black lines indicate the median over donors. See also Tables S1–S5.

The precursor definitions represented the set of Ab features that we hypothesized were necessary for an Ab to have strong potential to mature into a bnAb, with an Env-binding mode similar to a known bnAb. We hypothesized that a potential bnAb-precursor HC should share at least six characteristics with the bnAb: (1) HCDR3 length, (2) D gene identity, (3) D gene reading frame, (4) D gene position within the HCDR3, (5) V_H_ gene family (e.g. V_H_3 or V_H_4), and (6) J_H_ gene (Figures 2A and 2B and Table S2) (Steichen et al., 2019). Together these requirements ensured that potential precursors would share all or most germline-templated HCDR3 amino acids with the original (unknown) precursor. Thus, the major differences between potential and original precursors would be in the non-templated junction regions at the V-D and D-J boundaries. Using these precursor definitions as search criteria, we analyzed the frequency of five V2-apex bnAb classes in previously reported ultradeep immunoglobulin HC sequencing data from 14 HIV-naive human donors (Figures 2C and 2D and Table S3) (Steichen et al., 2019). We found PCT64 HC precursors in all 14 donors (100%) (Figure 2C) with a median frequency of 20.6 precursors per million BCRs (Figure 2D). For PG9/PG16, we found HC precursors in nine of 14 (64%) of donors, with median frequencies of 0.23 precursors per million BCRs among donors with at least one precursor and 0.135 precursors per million BCRs among all donors, lower than PCT64 by factors of 90 and 150, respectively (Figures 2C and 2D and Table S3). For the remaining classes, PGT/PGDM, CH01-CH04, and CAP256, we detected HC precursors in six (43%), two (14%), and one (7%) donor(s), respectively (Figure 2C), resulting in median precursor frequencies of zero computed among all donors in all three cases (Figure 2D and Table S3). For PGT/PGDM, the median frequency of HC precursors among donors with at least one precursor was 0.17 precursors per million BCRs, 121-fold lower than for PCT64. We also calculated precursor frequencies for paired heavy and light chains (“H+L” frequencies in Figure 2D) by scaling the HC precursor frequency by the frequency of the HC-light chain (LC) pairing obtained from paired NGS sequencing data (DeKosky et al., 2015).

To assess the degree of similarity between potential precursors and known bnAbs, we measured the number of mutations from each potential precursor HCDR3 to a known HCDR3 bnAb sequence (Figure 2E and Table S4). PCT64 precursors had an average of 14.2 of a possible 25 mutations from known PCT64 lineage bnAb sequences, and PG9/PG16 had an average of 18.4 out of a possible 30 mutations from mature PG9 or PG16 (Figure 2E and Table S4). For the PGT/PGDM class, which was detected in a smaller fraction of donors than PCT64 or PG9/PG16 but had a similar precursor frequency among positive donors as PG9/PG16 (Figures 2C and 2D), the precursors were a median of 26 mutations from a bnAb in the class, substantially further from a bnAb than PCT64 or PG9/16. Taken together, our analyses indicated that PGT/PGDM, CH01-CH04, and CAP256 classes are relatively poor vaccine targets due to their low precursor frequencies and, in some cases, high HCDR3 distances to bnAb. In contrast, our analyses indicated that PCT64 and PG9/16 are more suitable vaccine targets, with PCT64 having the most favorable precursor frequency and HCDR3 distance to bnAb.

### Multiple factors affect V2-apex bnAb precursor frequency

**Figure 3:**
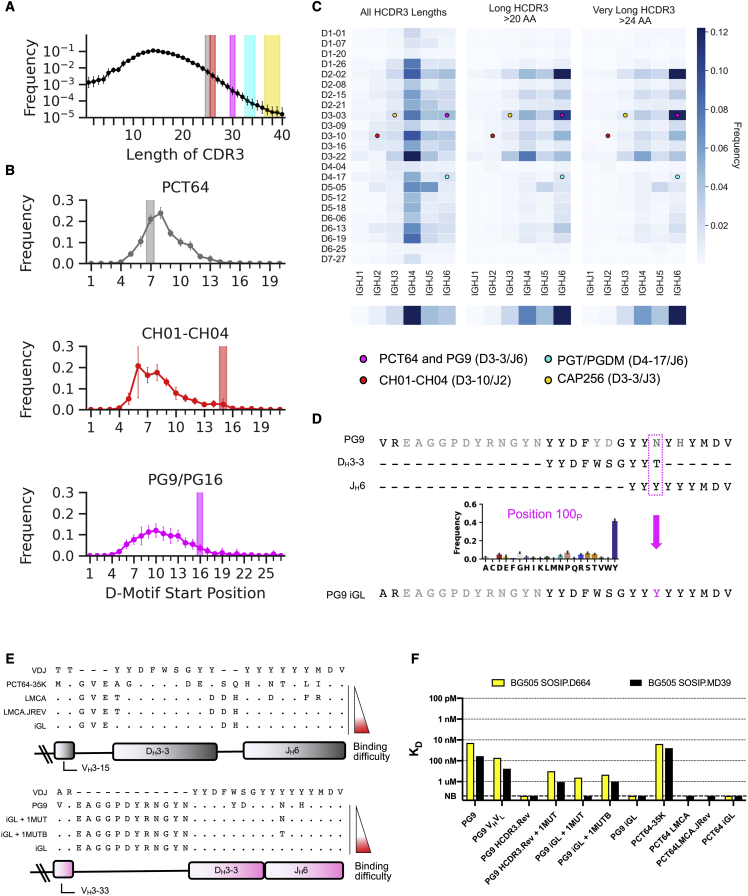
Multiple factors affect V2-apex bnAb precursor frequency (A) HCDR3 length distribution across all 14 donors, in which symbols represent the mean frequency across all donors, and error bars indicate the standard deviations. V2-apex bnAb precursor lengths are highlighted in colored boxes: PCT64, gray; CH04, red; PG9/PG16, magenta; PGT/PGDM, cyan; CAP256, gold. (B) Average D-gene motif start position within HCDR3 for the PCT64 motif in HCDR3s of length 25; the CH01-CH04 motif in HCDR3s of length 26; and the PG9/PG16 motif in HCDR3s of length 30; across the 14 donors. The highlighted colored bar indicates the start position of the D gene motif for each V2-apex bnAb. PGT/PGDM and CAP256 classes had insufficient matches to compute average D motif start positions. (C) DJ-gene usage frequency heat map among all HCDR3s, long HCDR3s (>20 amino acids), and very long HCDR3s (>24 amino acids) in 14 HIV-unexposed donors. The J gene frequency is shown in a single dimension at the bottom of each DJ heatmap. Points are shown for each DJ gene used for the V2-apex bnAbs. (D) PG9 DJ junctional analysis. The frequency of amino acids at position 100p, for all HCDR3s of length 30 with a PG9-like DJ junction. The most common amino acid at 100p was tyrosine, observed in multiple donors and clonotypes. (E) PG9 and PCT64 iGL variant junction alignments used in germline-targeting immunogen design. (F) PG9 and PCT64 iGL variants binding affinities measured by SPR against the native-like trimers BG505 SOSIP.D664 (Sanders et al., 2013) and BG505 SOSIP MD39 (Steichen et al., 2016). NB, no binding, is the highest concentration tested. See also Table S3.

To understand why precursor frequencies were significantly higher for PCT64 than for the other classes of V2-apex HCDR3-dependent bnAbs, we investigated the impact of several factors (HCDR3 length, VH family, D-gene motif start, and D-J pairing) on precursor frequency in our ultradeep HC sequencing dataset from 14 HIV-unexposed donors. For PGT/PGDM and CAP256, the primary contributor to a low precursor frequency was the HCDR3 length (Figure 3A). We calculated a mean HCDR3 length among all donors of 15.5 ± 4.8, in agreement with a previous analysis of eight of the 14 HIV-naive donors (Briney et al., 2019). HCDR3 lengths of 33 or 34, required for PGT/PGDM precursors, were only found at a frequency of 1.87 ×10^−4^ (187 BCRs per million BCRs), and HCDR3 lengths of 37–39, required for CAP256 precursors, were found at a frequency of 8.66 × 10^−5^ (74 per million B cell sequences). The precursor frequency for PGT/PGDM was also reduced disproportionately compared to the other classes (39-fold versus 2- to 4-fold), owing to PGT/PGDM using the less common V_H_1 and other classes using the more common V_H_3 (Table S3).

We next investigated the impact on precursor frequency due to the position of the D-gene sequence motif within the HCDR3. For all HCDR3s of length 25, required for PCT64, we calculated the frequency of the start position for the D-gene sequence motif “YDFWS” (Figure 3B). In PCT64 precursors, the “YDFWS” motif starts at position 7, which was the second-highest positional frequency given the D gene motif. For all HCDR3s of length 30, required for PG9/16 precursors, we calculated the frequency of the start position for the D-gene sequence “YDF” (Figure 3B). In PG9 and PG16, the “YDF” motif starts at position 16, whereas the most common start position for that motif is 10. The sub-optimal motif start position reduced the PG9/16 precursor frequency by a factor of 3.3. For CH01-CH04, which uses “YYGS” at position 15, the positional frequency for the D-gene motif was 8.3-fold lower than the ideal start position of 6. For PGT/PGDM and CAP256, we had too few sequences of the required length to produce a frequency for this metric.

Finally, we determined the D-J pairing frequencies for (1) all HCDR3s, (2) long HCDR3s (>20 amino acids), and (3) very long HCDR3s (>24 amino acids) (Figure 3C). PCT64 and PG9/PG16 both use D_H_3-3 and J_H_6, which are the most common D-J pairing for very long HCDR3s. D-J pairings for CH01-CH04 (D_H_3-10/J_H_2), PGT/PGDM (D_H_4-17/J_H_6), and CAP256 (D_H_3-3/J_H_3) were all found at low frequencies, especially among the long HCDR3 and very long HCDR3 subsets.

Taken together, these analyses indicated that the biggest factor contributing to diminished precursor frequency was HCDR3 length, especially for PGT145/PGDM and CAP256 classes. Although CH01-CH04 has a relatively shorter HCDR3 length, the D-gene placement within the HCDR3, along with an infrequently used J_H_2 gene, were the primary contributors to low precursor frequency. Use of the J_H_3 gene further reduced the CAP256 precursor frequency.

### Germline-targeting immunogens are needed

To determine whether engineering of a germline-targeting immunogen with affinity for PCT64 and PG9/16 precursors might be needed, we assessed the binding of mature and iGL variants of PCT64 and PG9 to native-like trimers BG505 SOSIP (Julien et al., 2013; Lyumkis et al., 2013; Sanders et al., 2013) and BG505 MD39 (Steichen et al., 2016). Previously reported PG9 iGL sequences used either asparagine (from the bnAb) or threonine (from the germline D gene) at position 100p (kabat numbering) (Medina-Ramirez et al., 2017; Sliepen et al., 2015), but our analysis of PG9-like junctions in our NGS data revealed that tyrosine was substantially more common than any other amino acid at 100p (Figure 3D). Therefore, we employed an updated PG9 iGL with tyrosine at 100p that likely better represents PG9-like precursors (Figures 3D and 3E). The iGL variants for PCT64 were the LMCA (PCT64 LMCA), a more reverted version of the LMCA in which the J_H_ gene somatic mutations were reverted to germline (PCT64 LMCA.JREV), and an iGL variant in which all unambiguous D and J gene templated mutations were reverted to germline (PCT64 iGL) (Figure 3E). We found that mature PCT64 bnAbs could bind BG505 native-like trimers, but the PCT64 LMCA, LMCA.JREV, and iGL antibodies had no detectable binding up to 5 μM (Figure 3E). Similarly, partially reverted PG9 variants (Figure 3E) could bind the native-like trimers, but PG9 iGL had no detectable binding (Figure 3F). The lack of detectable binding between BG505 native-like trimers and PCT64 LMCA, PCT64 iGL, and PG9 iGL indicated the need to engineer V2-apex germline-targeting (ApexGT) trimers with appreciable affinity for precursors.

### Structure-guided directed evolution produces V2-apex germline-targeting vaccine priming immunogens

**Figure 4:**
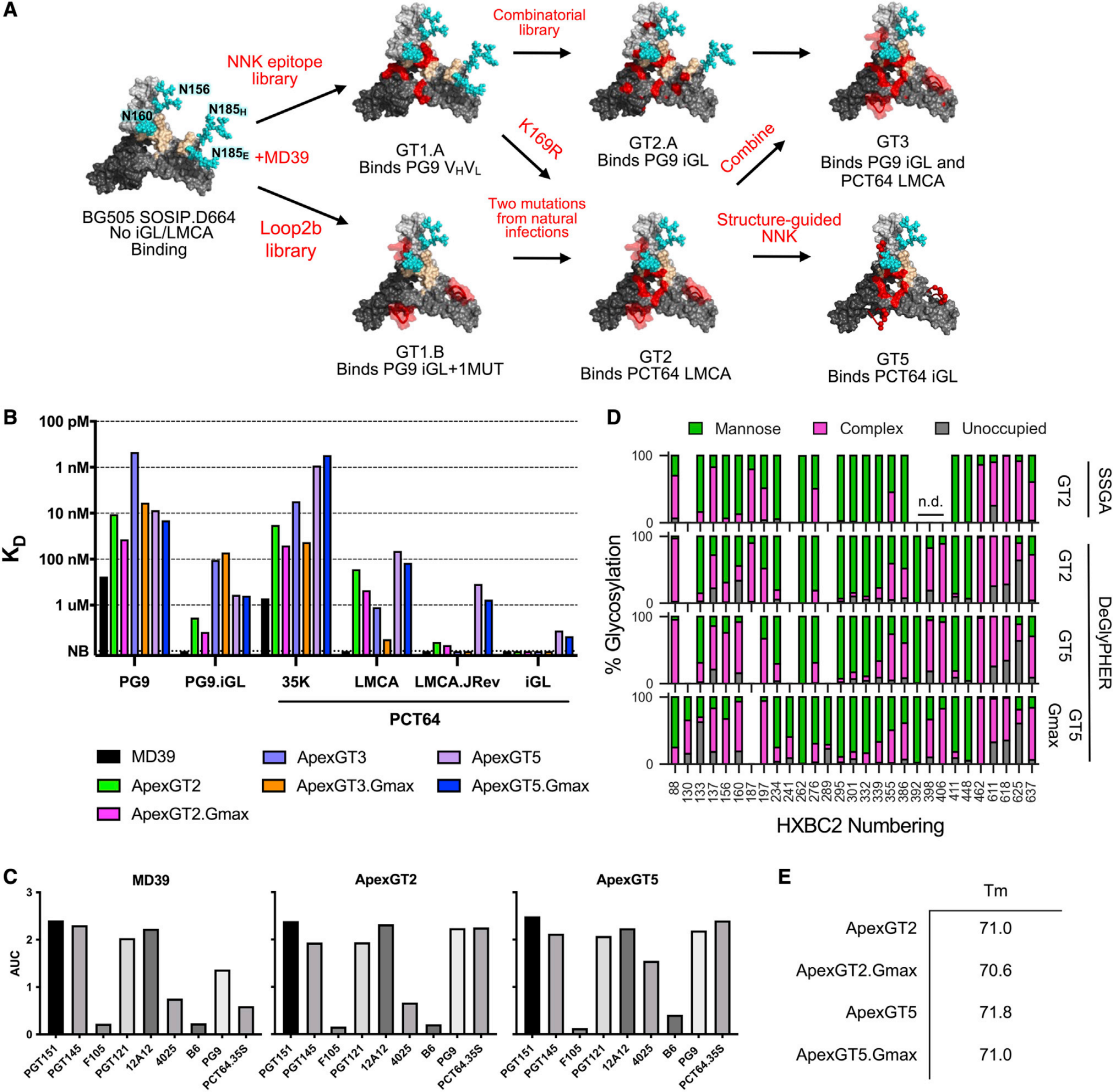
V2-apex germline-targeting immunogen design produces ApexGT trimers as candidate immunogens to prime PCT64 and PG9/16 responses (A) The immunogen design pathway starting from BG505 SOSIP.D664. The V1/V2 region is shown with remodeled glycans as cyan spheres. The PG9 binding site is beige and two of the three protomers are shown in shades of gray. The mutations determined from each library are shown in red surface patches. Loop2b (residues 181-191 in HXBC2 numbering) is shown as a red tube representation. The germline variant that bound the immunogen is shown below each step. For clarity, glycans are shown on only a single protomer. (B) SPR K_D_s for ApexGT trimer analytes binding to PCT64 and PG9 variants as IgG ligands with data fit using a 1:1 binding model. NB, no binding, is the highest concentration tested. (C) ELISA antigenic profiles of MD39 and ApexGT trimers. AUC is the area under the curve of the dilution series of the antibody shown on the x-axis. (D) Glycan composition for ApexGT trimers using two methods, SSGA (Allen et al., 2021) and DeGlyPHER (Baboo et al., 2021). High mannose, green; complex, pink; unoccupied, gray; N.D., glycan could not be resolved. (E) DSC melting temperatures of ApexGT trimers. See also Figures S1 and S2.

A germline-targeting priming immunogen should have both (1) appreciable affinity for bnAb precursors to enable precursor B cell activation and competitive fitness in germinal centers (GCs) and (2) higher affinity for corresponding bnAbs to provide an affinity gradient that may foster selection of mutations conducive for bnAb development (Jardine et al., 2013). To develop V2-apex germline-targeting immunogens with appreciable affinity for PCT64 and PG9 precursors and higher affinity for the corresponding bnAbs, we used mammalian cell-surface display-directed evolution with a BG505 SOSIP.D664 native-like trimer platform. Because PG9 iGL, PCT64 LMCA, and PCT64 iGL had no detectable affinity for either BG505 SOSIP.D664 or BG505 MD39 (Figures 3E and 3F), we used a “bootstrapping” approach (Steichen et al., 2016) starting with partially reverted PG9 and PG16 variants for screening against multiple library types (Figure 4A).

We generated two NNK-type libraries (degenerate codons that encode all 20 amino acids with 32 codons), an NNK-scanning library, and an NNK-combinatorial library. The NNK-scanning library contained sequential NNK codons at positions 32-185_H_ (HXBC2 numbering) as described previously (Kulp et al., 2017; Steichen et al., 2016) (Figure S1). The NNK-combinatorial library contained NNK combinations at contact residues between PG9 and BG505 SOSIP.D664, K170-V173. These contact residues were identified by docking PG9 into the BG505 SOSIP crystal structure using the modeling suite Rosetta (Das and Baker, 2008). The NNK-scanning library showed enrichment after sorting with a fully reverted PG16 HCDR3 but a mature V_H_/V_L_ (PG16-HCDR3_Rev_). The NNK-combinatorial library showed enrichment after sorting with mature and partially reverted variants of PG9/PG16 (Figure S2A). The enriched mutations for both libraries were combined and expressed on the background of MD39, a variant of BG505 SOSIP.D664 with improved stability, expression, and antigenicity (Steichen et al., 2016), yielding ApexGT1.A, a trimer with enhanced affinity to PG9 V_H_V_L_ and PG9 iGL+1MUT (Figures 4A, S2B, and S2C). A second-generation combinatorial library combining enriched mutations from the first two libraries was sorted with fully and partially reverted PG9 and negatively sorted against the V3-directed non-neutralizing Ab 4025 to enrich for sequences of well-formed, “closed” trimers. This yielded ApexGT2A, a trimer with enhanced affinity to PG9 germline variants and with detectable affinity for fully reverted PG9 iGL (Figures S1 and S2C).

We also pursued a library of Loop2b (D180–Y191) because it was implicated as a major contact with PG9 in our Rosetta model (Figure S1). To generate the Loop2b library, we considered all natural sequences gathered from the Los Alamos National Laboratory (LANL) HIV Sequence Database (http://www.hiv.lanl.gov/). This search yielded 1.1 × 10^4^ unique Loop2b sequences, which we synthesized and cloned onto BG505 SOSIP.D664 to create a library for mammalian display. We sorted this library using mature, partially reverted, and fully reverted PG9 iGL fragment antigen-binding domains (Fabs) and again used negative selection by Ab 4025 to enrich for “closed” trimers. These efforts yielded ApexGT1B, a trimer that bound PG9 iGL + 1MUT and enhanced affinity for mature PG9 (Figure S2C).

To improve binding to PCT64 bnAbs and rescue binding to PCT64 LMCA, we combined the enriched loop sequence from ApexGT1.B, the K169R mutation from ApexGT1A, and two naturally occurring mutations from the PCT64 donor, M161A and D167N (Landais et al., 2017). The resulting construct, ApexGT2, bound to PCT64 LMCA with a monovalent affinity of 167 nM and had 25-fold and 39-fold enhanced affinity for PG9 and PCT64.35K, respectively, compared to MD39 (Figures 4B and S2C). By enzyme-linked immunosorbent assay (ELISA) antigenic profiling, we found that ApexGT2 had a similar profile to MD39 (Figure 4C). By glycan composition analysis, ApexGT2 had an overall similar glycan profile to BG505 SOSIP.D664 (Behrens et al., 2016; Cao et al., 2017, 2018) across two separate glycan analysis techniques, single-site glycan analysis (SSGA) (Allen et al., 2021) and DeGlyPHER (Baboo et al., 2021). Position 187 within Loop2b was not glycosylated in MD39 but was well occupied in ApexGT2 and composed mainly of complex-type glycans (Figure 4D). ApexGT2 also showed good thermal stability by differential scanning calorimetry (DSC), with a T_m_ of 71°, reduced by 6° from MD39 but still within the range for BG505 SOSIP.D664-based native-like trimers (Kulp et al., 2017; Sanders et al., 2013).

The third-generation trimer, ApexGT3, was a combination of ApexGT2A and ApexGT2. ApexGT3 showed improved PG9 iGL binding, with a K_D_ of 104 nM. However, the affinity for PCT64 LMCA was reduced by a factor of 7 compared to ApexGT2, necessitating the need for further engineering. To improve PCT64 LMCA affinity while maintaining PG9 iGL affinity, we engineered ApexGT2 with three combinatorial-NNK libraries at contact positions identified at the antigen-antibody interface in the ApexGT2-PCT64 LMCA structure (described in the next section). PCT64 LMCA was used as a positive probe to select mutations that improve binding affinity, while the non-neutralizing CD4-binding-site-directed B6 Fab was used as a negative probe to select for “closed” trimers. Enriched mutations were added to ApexGT2 to yield ApexGT5, our most advanced GT trimer with the best affinity profile to date. ApexGT5 had K_D_s of 66 nM and 0.9 nM for PCT64 LMCA and PCT64.35K bnAb, respectively, and K_D_s of 596 nM and 8.6 nM for PG9 iGL and mature PG9, respectively (Figures 4B and S2C). Notably, ApexGT5 was the first of our GT trimers to acquire measurable affinity for PCT64 iGL, a substantially superior model precursor compared to the LMCA. The K_D_ for PCT64 iGL was 3.6 μM, within the range of affinities capable of stimulating responses from rare precursors using multivalent immunogens (Abbott et al., 2018; Huang et al., 2020; Kato et al., 2020; Wang et al., 2021). The binding affinity of ApexGT5 for PCT64 LMCA.JREV, also superior to LMCA as a model precursor, was 347 nM, 18-fold higher than for ApexGT2 (Figures 4B and S1). Thus, ApexGT5 had appreciable affinities for PCT64- and PG9-inferred precursors and higher affinities for the corresponding mature bnAbs. However, we have not detected ApexGT5 affinity to a select set of PCT64 or PG9/PG16 NGS precursors (Table S5). The overall antigenic profile for ApexGT5, measured by ELISA, was in good agreement with MD39, except for elevated binding to the V3 non-nAb 4025 (Figure 4C), and the melting temperature remained high, at 71.8°C (Figure 4D), both of which suggested an overall native-like structure albeit with a slightly more exposed V3 loop.

To minimize “holes” in the glycan shield and focus antibody responses to the V2-apex epitope, we added the N130 glycosylation site, which was implicated in shaping the PCT64 lineage (Rantalainen et al., 2018) and N241 and N289 glycosylation sites, which are conserved across most HIV isolates but absent from the BG505 isolate (McCoy et al., 2016) (Figure S2B). Adding these three glycosylation sites to ApexGT2 and ApexGT5, producing ApexGT2.Gmax and ApexGT5.Gmax, respectively, only modestly reduced affinity for PG9 and PCT64 variants tested and modestly decreased thermostability (Figures 4B and 4E and S2C). Glycosylation of ApexGT5 and ApexGT5.Gmax (Figure 4D) was broadly similar to BG505 SOSIP except for substantially elevated levels of complex glycans at positions 156, 160, and 197 (Behrens et al., 2016; Cao et al., 2017, 2018).

### Structures of ApexGT trimer-Fab complexes determine trimer interactions with precursors and mature bnAbs for both PCT64 and PG9

To gain structural insight into the capacity of ApexGT trimers to bind V2apex precursors with appreciable affinities and mature bnAbs with higher affinities, we determined single-particle cryo-EM structures of ApexGT trimers in complex with the Fabs of both PCT64 LMCA and PG9 iGL as well as their mature broadly neutralizing counterparts, PCT64 35S and PG9. We determined the structure of PCT64 LMCA complexed with a version of ApexGT2 called ApexGT2.2MUT that had the Loop2b glycan at N187 knocked out (T189A) and an N195D mutation that modestly improved the affinity for PCT64 LMCA (K_D_ of 78 nM compared to a K_D_ of 167 nM for ApexGT2 [Figure S2C]). We also determined structures for PG9 iGL in complex with ApexGT3 and for mature PG9 in complex with a Q130N variant, ApexGT3.2MUT, that contained a glycosylation site at position 130. A summary of all cryo-EM structures can be found in Table S6.

### ApexGT2-PCT64 variant structures reveal complex interactions, with subtle differences between bnAb and precursor

**Figure 5:**
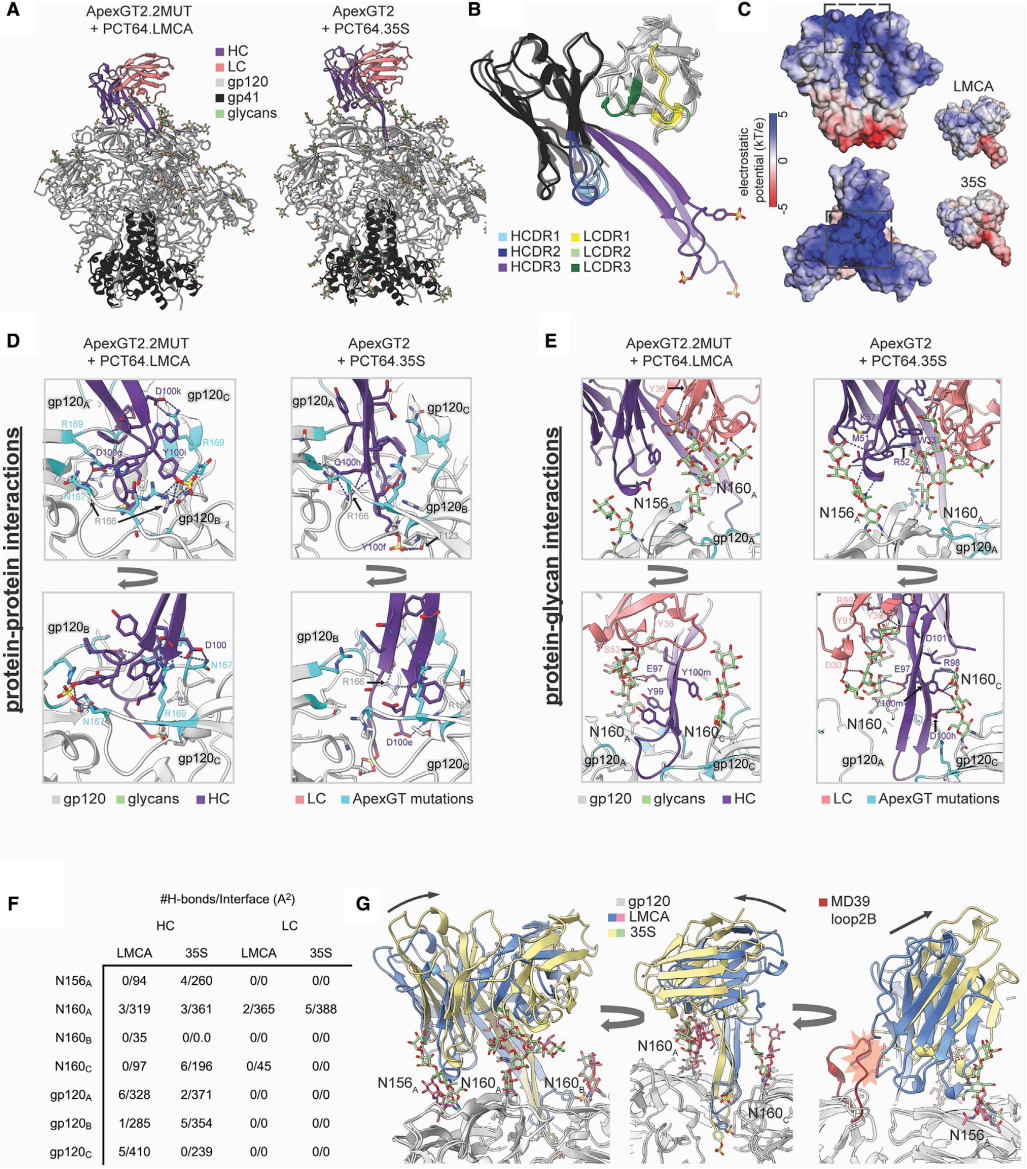
Cryo-EM structures of ApexGT2.2MUT bound to PCT64 LMCA Fab, and ApexGT2 bound to PCT64 35S Fab, reveal complex interactions to guide design of PCT64-targeting ApexGT trimers (A) Refined atomic models of both complexes. (B) Isolated structure and domain organization of both Fabs aligned to their HCs, with PCT64 35S shown as partially transparent. (C) Electrostatic potential surfaces of both Fabs and of ApexGT2.2MUT (without glycans). (D) Close-up views of the binding interface showing antibody-gp120 protein interactions for both complexes. The outset shows an additional h-bond between the LMCA HC and gp120 not visible in the close-up. (E) Same as (D) but showing antibody-glycan interactions, with some parts of the LC hidden to enable viewing of specific residue contacts. (F) Table showing the number of h-bonds and total interfacial surface area between the different components of the epitope/paratope of both complexes. (G) Both complexes aligned on gp120A with arrows indicating the change in binding angle from LMCA to 35S. The additional red fragment on the rightmost panel is a model of the native BG505 SOSIP.MD39 loop2B highlighting the potential steric clashes with the LMCA HC. See also Table S6 and Figures S2–S5.

The structures of ApexGT2.2MUT bound to PCT64 LMCA and ApexGT2 bound to PCT64 35S (Figures 5A, S3, and S4) both showed the expected 1:1 stoichiometry and tilted angle of approach relative to the 3-fold axis as observed previously for autologous PCT64 complexes (Landais et al., 2017; Rantalainen et al., 2018), as well as the extended beta-hairpin conformation of the HCDR3 domain as it protrudes into the trimer (Figure 5B). In the previously reported apo crystal structure of a PCT64 LMCA variant (with three light-chain mutations that confer neutralization to autologous viruses), the HCDR3 formed a collapsed coil (Rantalainen et al., 2018). Here, we solved the apo crystal structure of the true LMCA Fab and found a similar collapsed coil/disordered HCDR3 conformation (Figures S5A–S5C). In our PCT64 LMCA complex structure, the HCDR3 adopted a beta-hairpin conformation resembling more mature PCT64 antibodies (e.g., PCT64 13C; Figure S5D), which suggested that either the apo coil conformation was a crystallization artifact or that PCT64 LMCA binds via an induced-fit mechanism. The HCDR3 of the mature bnAb PCT64 35S in complex with ApexGT2.2MUT showed only minor differences relative to the apo crystal structure of PCT64 35S Fab (PDB: 6CA6), adopting a slightly straighter beta-hairpin conformation (Figure S5E).

Sulfated tyrosines, which play a critical role in PG9/PG16 (Pejchal et al., 2010), were resolved in the EM map density for both PCT64 LMCA (Y100e and Y100i) and 35S (Y100f) (Figure 5B) (Landais et al., 2016). Electrostatic potentials calculated with the Adaptive Poisson-Boltzman Sampling (APBS) algorithm (Jurrus et al., 2018) showed the positive and negative electrostatic potential of the ApexGT trimer binding surface and PCT64 HCDR3 domains, respectively (Figure 5C). This electrostatic complementarity likely contributes to the binding affinity.

We next examined protein-protein interactions between ApexGT mutations and both PCT64 LMCA and PCT64 35S (Figure 5D). R169 was found to engage the LMCA through hydrogen-bonding interactions on all three gp120 protomers, and N167 was found to engage two of the three protomers. R166, native to the BG505 isolate, was positioned in two of three protomers for potential cation-π interactions with the aromatic HCDR3 residues W100b and F100a. The PCT64 LMCA interactions with GT mutations R169 and N167 (Figure 5D) were not found in the 35S complex, but the interactions with R166 were retained by 35S. The more extended HCDR3 conformation in the 35S complex positioned the sulfated Tyr100f residue deeper within the apex where it formed multiple new h-bonds with two protomers (Figure 5D).

In addition to engaging gp120 through protein-protein interactions, both PCT64 LMCA and 35S engage apex-related glycans, in particular the N160gp120_A_ glycan buried in the HC/LC interface (Figures 5E and S5F). Mature 35S forms a larger number of specific h-bonds with the N160 and N156 glycans on gp120_A_ (Figure 5F). These interactions are likely dynamic, as 3-D variability analysis (Punjani and Fleet, 2021) revealed flexibility of both Fabs relative to the trimer apex involving shifting contacts with the N156gp120_A_ glycan (Video S1).

Movie S1. 3-D variability analysis of PCT64 lineage Fabs reveals flexibility relative to trimer and shifting glycan contacts, related to Figure 5 3-D variability analysis movies depicting heterogeneity along the first three eigenvectors for PCT64.LMCA and PCT64.35S

Finally, using a subset of apex-unliganded trimers identified from the PCT64 35S complex dataset, we obtained a 2.7-Å-resolution reconstruction of ApexGT2 with unliganded trimer apex (Figures S4C–S4F), from which we determined how each glycan was being displaced in the complex relative to its average apex-unliganded position. We found that three glycans, N156gp120_A_, N160gp120_A_, and N160gp120_c_, were displaced less by the 35S Fab than by the LMCA (Figure S5G), which is indicative of better glycan accommodation by 35S. This improved accommodation is a direct result of the steeper angle of approach of 35S relative to the trimer apex (Figure 5G). The steeper angle of approach also relieves potential clashes with longer Loop2b regions, such as the one from wild-type BG505 (Figure 5G). The conformation of Loop2B in both apo and PCT64-35-bound trimers, and the lack of clear map density for the N187 glycan, both suggest that the tip of the loop is predominantly oriented away from the 3-fold axis and that the glycan itself is highly flexible, consistent with the N187 glycan having negligible effect on K_D_ (Figure S2C). Overall, these structures revealed complex trimer-binding interactions for PCT64 LMCA and 35S, involving protein-protein and protein-glycan contacts, and showed that electrostatics, hydrogen bonds, cation-π interactions, potential induced fit of HCDR3, and displacement of glycans all likely contribute to the affinities and binding poses with tilted angles of approach. The LMCA structure provided guidance for the design of ApexGT5, as described above, and both structures will provide guidance for design of further improved priming immunogens, as well as boosting immunogens.

### ApexGT3-PG9 variant structures show that PG9 precursor and bnAb have similar angles of approach and HCDR3 conformations

**Figure 6:**
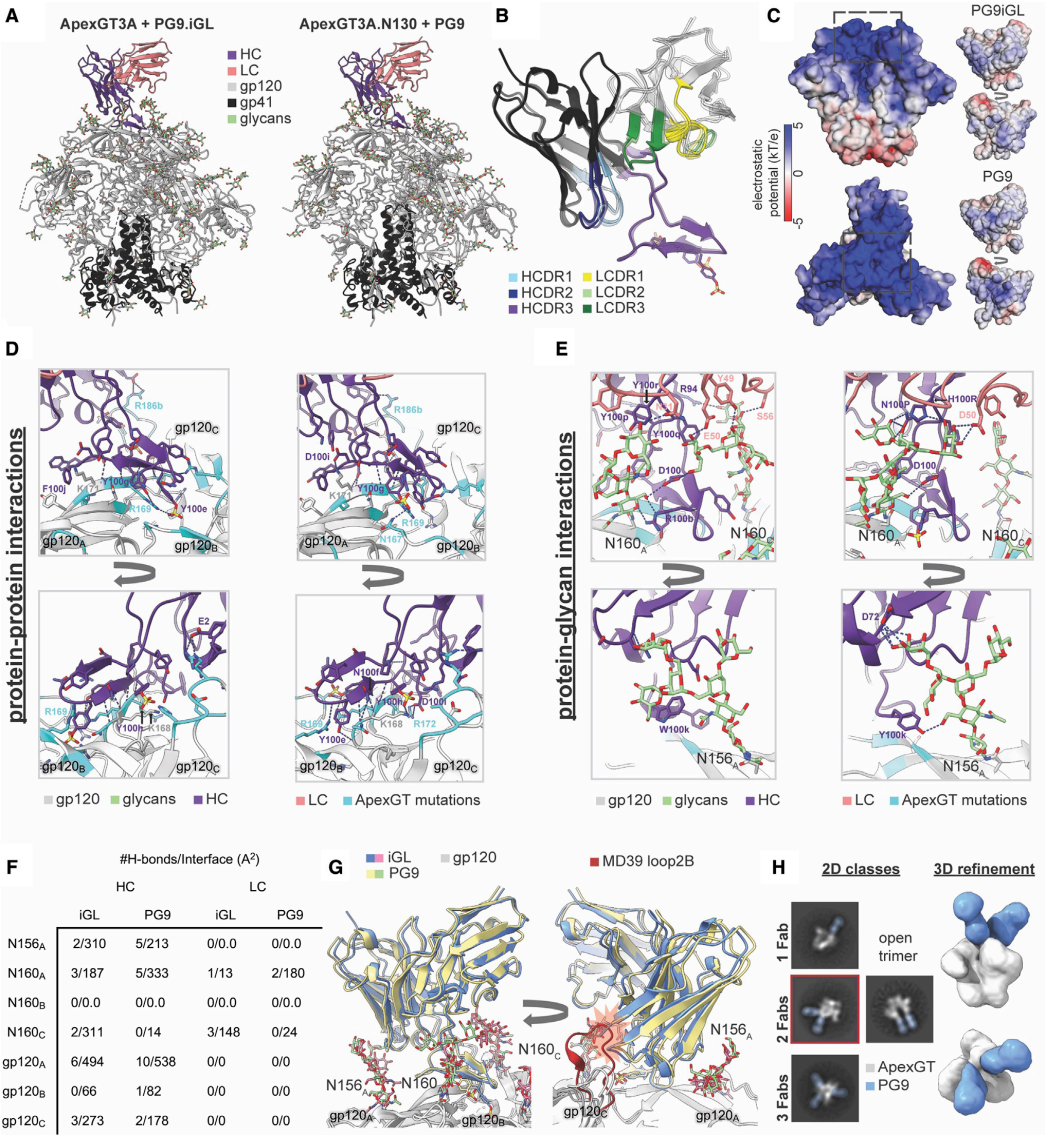
Cryo-EM structures of ApexGT3A bound to PG9 iGL Fab, and ApexGT3A.N130 bound to PG9 Fab, reveal complex interactions to guide design of PG9-targeting ApexGT trimers (A) Refined atomic models of both complexes. (B) Isolated structure and domain organization of both Fabs aligned on their HCs, with PG9 shown as slightly transparent. (C) Electrostatic potential surfaces of both Fabs and of ApexGT3A (without glycans) calculated with APBS. (D) Close-up views of the binding interface showing antibody-gp120 protein interactions for both PCT64 complexes. All gp120 residues within 4Å of the HCDR3 are shown and h-bonds are indicated with dashed blue lines. (E) Same as (D) but antibody-gp120 glycan interactions. (F) Table showing number of h-bonds and total interfacial surface area between the different components of the epitope/paratope of both complexes. (G) Both complexes aligned on gp120A revealing an identical binding angle. The additional red fragment on the rightmost panel is a model of the native BG505 SOSIP.MD39 loop2B highlighting the potential steric clashes with the HC of both Fabs. (H) NSEM 2-D class averages of ApexGT3 in complex with PG9 showing classes with more than one Fab bound (Fabs false-colored blue) along with a segmented 3-D reconstruction of the 2 Fab bound class (highlighted in red). See also Table S6 and Figures S5–S7.

The structures of ApexGT3 bound to PG9 iGL, and ApexGT3.2MUT bound to PG9, shared characteristics with the PCT64 complexes described above. Both PG9 Abs engage the GT trimer with 1:1 stoichiometry, bind at a tilted angle of approach relative to the trimer apex (Figure 6A), use an extended anionic HCDR3 containing sulfated tyrosines (Figures 6B–6D), and form extensive interactions with apex glycans (Figures 6A–6C). The additional glycans at positions N130 and N185_H_, present on ApexGT3.2MUT in the PG9 complex but not on ApexGT3 in the PG9 iGL complex, do not make direct contacts with the PG9 Fab. In contrast to PCT64 LMCA, PG9 iGL relies heavily on beta-sheet interactions with the C-strand of V2 on gp120_A_, does not reach as far into the positively charged 3-fold symmetry axis (Figure 6D), and utilizes a less negatively charged HCDR3 (Figure 6C).

The beta-sheet main-chain interactions with the gp120 C strand are in good agreement with the previously reported crystal structure of PG9 bound to a V1/V2 protein scaffold (McLellan et al., 2011) (Figure 6D). The GT mutation R169 forms h bonds with the HCDR3 on two different gp120 protomers and is also positioned well for co-planar cation-π interactions with Y100e on both PG9 and PG9 iGL. The sulfated tyrosine at position 100e on PG9 iGL interacts with R169 on gp120_A_, while the mature PG9, which includes a sulfated Y100g, can also engage the N167gp120_A_ glycan.

Similar to the PCT64 complexes, the number of interactions with the glycan at N160gp120_A_ increases with maturation, with PG9 gaining four h-bonds with distal mannose residues (Figures 6E and 6F). Specifically, the germline-encoded tyrosines at both 100p and 100r in PG9 iGL, but not in PG9, displace N160gp120_A_ out of the HC/LC binding pocket (Figure 6E) and likely contribute to the poor binding affinity of the iGL for most native Env trimers. In addition to the interactions with the N160gp120_A_ glycan, PG9 also acquires additional h-bonds with the N156g120_A_ glycan (Figure 6E), one of which arises from the W(100k)Y mutation in the HCDR3. PG9 iGL, but not PG9, engages with the N160gp120_C_ glycan, using both the HC and LC (Figure 6E), indicating that a trimeric interface is necessary to engage iGL but not mature PG9. Indeed, evidence from negative stain EM (NSEM) suggests that two or three PG9 Fabs can bind a single ApexGT3 trimer (Figure 6H), but due to steric hinderance the trimer would likely need to adopt an open conformation to accommodate the third Fab, as seen in some of the negative stain 2-D classes (Figure 6H).

PCT64 LMCA and PG9 iGL show near-identical binding angles with their respective ApexGT trimers (Figure S5I). This tilted binding angle could potentially cause clashes with the elongated Loop2b of BG505-derived trimers. Our structures reveal that this clash is avoided by the shorter Loop2b of both ApexGT trimers (Figures 5G and 6G).

Inter-protomer distance measurements between gp120 subunits at the 3-fold symmetry axis indicate an asymmetric “swelling” of the V2-apex binding site upon antibody binding (Figure S5J). This swelling only occurs upon binding for V2-apex bnAbs and is not observed for other classes of bnAbs. In addition, ApexGT trimers have a lower thermal stability (∼71°C, Figure 4E) than MD39 (77°C, [Steichen et al., 2016] as well as higher V3 reactivity than MD39 [Figure 4C]), which may be indicative of reduced stability in the apex interface. This corroborates a previous hypothesis that an intrinsic lability of the trimer apex may be necessary for binding to V2-apex bnAbs (Lee et al., 2017). Overall, the trimer-Fab structures for PG9 and PG9 iGL revealed interactions of similar complexity as in the PCT64 trimer-Fab structures, including both protein-protein and protein-glycan interactions, and provided a structural understanding for how the ApexGT trimer gained affinity for the precursor. The angle of approach and HCDR3 conformation were quite similar for precursor and bnAb in the PG9 complexes but differed in the PCT64 complexes, which implied that bnAb structural maturation may be simpler for PG9 than for PCT64.

### mRNA-delivered membrane-bound ApexGT trimers are lead priming immunogens

**Figure 7:**
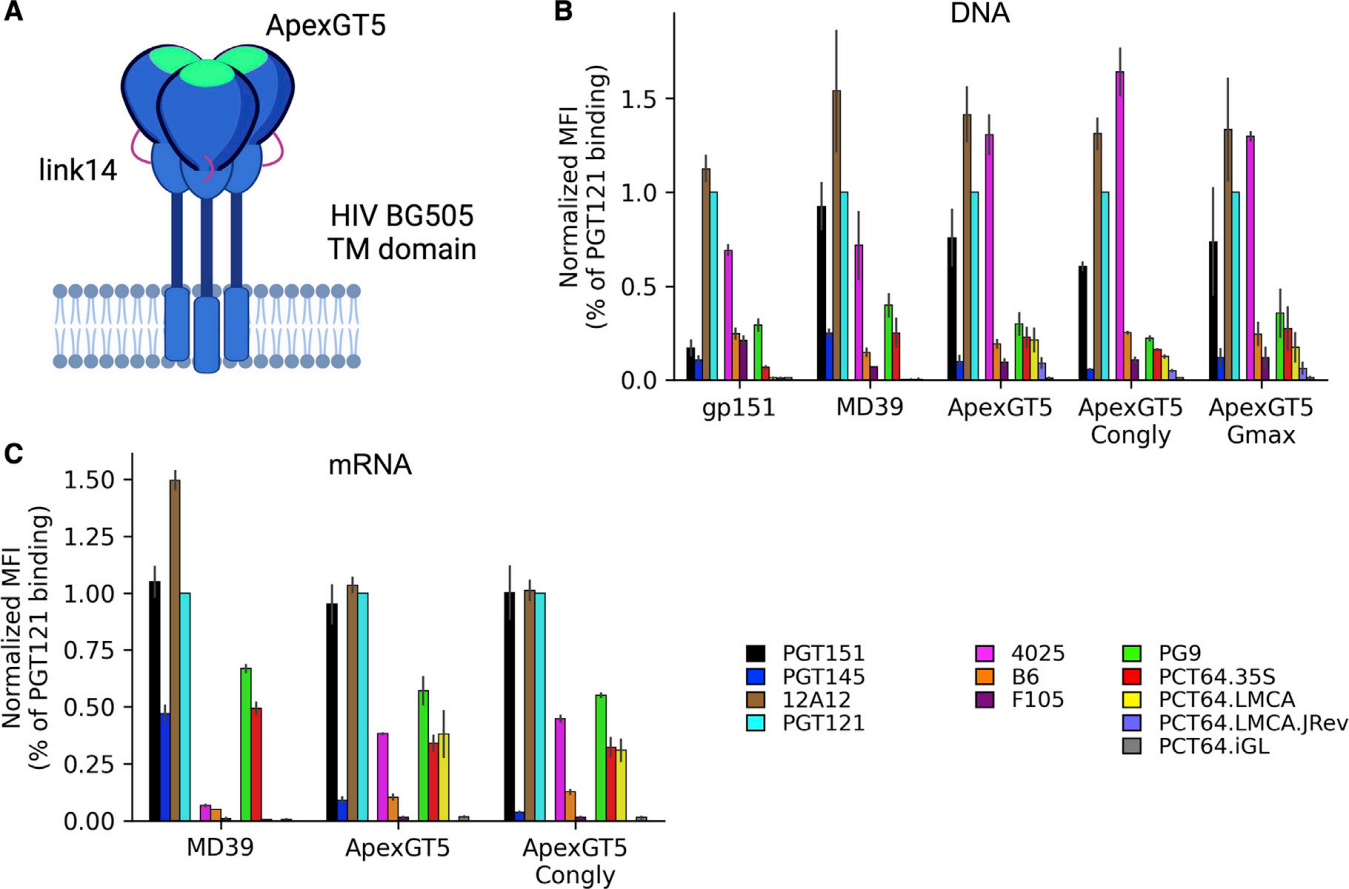
Developing membrane-bound ApexGT trimers produces candidates for nucleic acid delivery (A) Cartoon schematic of cell-surfaced displayed ApexGT5 trimer. Link14, shown in pink, bridges gp41 and gp120. Location of GT mutations is indicated in green. (B) Cell surface antigenic profile for DNA-expressed membrane-anchored trimers binding to IgG for control bnAbs (quaternary, PGT151 and PGT145; CD4bs, 12A12; and V3-glycan, PGT121), non-nAbs (V3, 4025; CD4bs, B6 and F105), V2-apex bnAbs (PG9, PCT64), and V2-apex bnAb precursors (PCT64.LMCA, PCT64.LMCA.JREV, PCT64.iGL). Mean-fluorescence intensity (MFI) via fluorescence-activated cell sorting (FACS) binding was normalized to PGT121 binding with error bars representing standard deviation (n = 2). All trimers are based on the BG505 isolate, and all have a c-terminal truncation at residue 709. gp151 contains no other modifications. MD39 contains stabilizing mutations in BG505 SOSIP MD39 (Steichen et al., 2019). ApexGT trimers contain GT mutations described in the text. (C) Similar to (B) but with membrane-anchored trimers expressed from mRNA.

The rapid development and high efficacy of the mRNA-based coronavirus disease 2019vaccines (Baden et al., 2021; Polack et al., 2020), which were based on delivery of mRNA coding for membrane-anchored full-length spike proteins, suggests that a similar approach to delivering HIV trimers (Figure 7A) could potentially benefit HIV vaccine development (Aldon et al., 2018). Fully native HIV trimers are anchored to a lipid membrane on the virus or an infected cell, but most HIV vaccine discovery efforts have utilized trimers in a soluble format owing to the difficulties associated with production and purification of membrane proteins. mRNA delivery of membrane-bound trimer immunogens would (1) at least partially occlude the trimer base that when exposed on soluble trimers is immunodominant and elicits non-neutralizing and trimer-degrading antibodies (Bianchi et al., 2018; Cirelli et al., 2019; Hu et al., 2015; Nogal et al., 2020; Turner et al., 2021), (2) offer potentially highly multivalent *in vivo* trimer presentation on microvesicles or exosomes (Bansal et al., 2021) or cell surfaces, and (3) potentially be advantageous for trimer quaternary conformational sampling (Walker et al., 2009) and glycosylation (Cao et al., 2018).

We sought to develop an mRNA vaccine platform encoding membrane-bound ApexGT trimer immunogens. Initial *in vitro* transfection experiments with DNA coding for membrane-bound BG505 MD39 native-like trimers used a 14-amino-acid linker in place of the furin cleavage site (link14) to eliminate the need for a furin cleavage (Steichen et al., 2016, 2019) and tested two different truncations of the gp160 C terminus. We found that the shorter construct, termed MD39 link14 gp151 and truncated at residue 709 (C terminus, VIHRVR), had 8-fold superior cell-surface expression compared to the longer construct that contained a known endocytosis motif (GYXXØ) and was truncated at residue 716 (C terminus, RVRQGYSPLS) (data not shown). Rabbit immunization experiments indicated that mRNA membrane-bound MD39 trimers elicited reduced base-directed responses and similar or better autologous neutralizing responses compared to mRNA- or protein-delivered soluble MD39 trimers (W.R.S., unpublished data). We therefore developed ApexGT5 membrane-bound trimers utilizing the link14 linker and gp151 C-terminal truncation. Cell-surface antigenic profiling of DNA-transfected cells showed that, relative to the initial gp151 construct, the MD39 construct had stronger binding to trimer-specific bnAbs PGT151, PGT145, and PCT64 35S and weaker binding to non-neutralizing mAbs 4025, B6, and F105, indicating that MD39 gp151 had a more native-like trimer structure than the initial gp151 (Figure 7B). ApexGT5 had a similar overall antigenic profile to MD39 except with modestly elevated binding to the non-neutralizing V3-directed Ab 4025; reduced binding to PGT145, PG9, and PCT64 V2-apex bnAbs; and increased binding to PCT64 LMCA and PCT64 LMCA.JREV (Figure 7B). Antigenicity of the "congly" variant (with N241 and N389 glycosylation sites restored) and Gmax variant of ApexGT5 were similar to ApexGT5 (Figure 7B). To enable antigenicity evaluation from mRNA-transfected cells, and eventual immunogenicity testing (Melzi et al., 2022), we synthesized Moderna mRNAs for MD39 gp151, ApexGT5 gp151, and ApexGT5 congly gp151. The overall antigenic profiles of ApexGT5 and MD39 from mRNA-transfected cells were similar to those from DNA-transfected cells, with ApexGT5 having a similar overall profile to MD39 except for reduced PGT145, increased Ab 4025, and increased PCT64 LMCA and PCT64 LMCA.JREV binding (Figure 7C). We concluded that ApexGT5 gp151 and ApexGT5 congly gp151 represented promising candidates to be tested for priming of PCT64 and potentially PG9 precursor responses by mRNA delivery.

## Discussion

HIV bnAbs to the V2-apex epitope are important leads for vaccine design, but they present significant challenges for vaccine elicitation due to the dependence of their neutralizing activity on long HCDR3s. The first requirement for a vaccine to elicit a V2-apex bnAb will be to consistently prime potential bnAb-precursor B cells with genetic properties similar to the bnAb, including a long HCDR3. In general, HCDR3 sequences are diverse among precursors for any one class of bnAb, owing to junctional diversity of non-templated regions of HCDR3s. Thus, to define precursors for any particular class of bnAb, shared features must be defined that can be found in the shared human naive (or memory) B cell repertoire. We previously developed a generalized germline-targeting vaccine design strategy for HCDR3-dominant antibodies, a strategy that leverages ultradeep sequencing data from the human HC B cell repertoire and attempts to design immunogens that can bind diverse HCDR3s belonging to any single bnAb class (Steichen et al., 2019). Here we began adapting that strategy to the exceptionally long HCDR3s for V2-apex class bnAbs by performing a frequency analysis for HCDR3-dominant V2-apex bnAb precursors in ultradeep sequencing data from 14 healthy donors. Our analysis led us to prioritize PCT64 and PG9/16 for V2-apex vaccine targeting, with PCT64 of highest priority, owing to higher precursor frequencies and shorter HCDR3 mutational distances to a known bnAb. PCT64 has these favorable properties because a large portion (20 of 25 amino acids) of its HCDR3 is templated by D or J genes. Based on our analyses, we hypothesize that germline-targeting vaccine design for long HCDR3 bnAbs will generally be more favorable for bnAbs with larger fractions of their HCDR3s templated. Conversely, bnAbs with large untemplated regions of HCDR3 will be difficult targets because precursors with shared features within those untemplated regions will be very rare. In general, the population frequencies of the templated D and J gene alleles will also be an important factor in precursor frequency.

We engineered ApexGT trimer immunogens with affinity for precursors to both PCT64 and PG9/16, determined four high-resolution cryo-EM structures of ApexGT trimers with bnAb and precursor forms of PCT64 and PG9, and illustrated how one of the early-determined structures (ApexGT2+PCT64 LMCA) was employed to guide design of a higher-affinity ApexGT trimer (ApexGT5).

From our PCT64 cryo-EM structures, we found that maturation of the PCT64 lineage was achieved through improved accommodation of apex-associated glycans, primarily N160 and N156 on gp120 protomer A; more extensive and less specific interaction between the HCDR3 and gp120 V2/V3 loops on gp120 protomer A and B; and reduced clashes with loop2B and loop2B glycans on gp120 protomer C. All these changes were made possible by the extension and rigidification of the beta-hairpin, which allowed the sulfated tyrosine at the tip of the HCDR3 loop to reach deeper in 3-fold axis binding pocket and created a steeper angle of approach relative to the trimer apex. Thus, in order to achieve similar maturation through sequential prime-boost vaccination, boosting immunogens will likely need to have fewer positively charged residues in the V2 loop and longer, more glycosylated loop2Bs. Taken together, our PG9 structures revealed that maturation of this lineage was achieved primarily through improved interactions with apex-associated glycans N160 and N156 on gp120 protomer A, similar to PCT64, and somewhat surprisingly, reduced interaction with the N160 glycan on gp120 protomer C. Unlike PCT64, the PG9 HCDR3 loop structure and its interactions with gp120 protein residues are more conserved between the iGL and mature antibodies, and there is no change in the angle of approach relative to the trimer apex. Thus, the maturation path from precursor to bnAb appears to be simpler for PG9 than for PCT64. That finding, together with the higher potency of PG9/16, encourages continued work to develop optimal priming immunogens for PG9/16, despite their lower precursor frequency compared to PCT64.

We concluded this work by developing membrane-bound forms of our ApexGT trimers that are promising for mRNA vaccine delivery, characterizing them by cell-surface antigenic profiling from both DNA and mRNA transfection. In a companion paper, we and colleagues report immunogenicity studies of ApexGT trimers delivered as soluble proteins and as mRNA-lipid nanoparticles (LNPs) encoding membrane-bound trimers (Melzi et al., 2022). These studies represent promising steps toward development of a germline-targeting immunogen capable of priming V2-apex precursor B cells in humans.

### Limitations of the study

This study has several limitations. Our immunoinformatic analyses were based on human immunoglobulin ultradeep sequencing data from 14 humans; the generality of the analysis would be strengthened if additional ultradeep sequencing datasets could be analyzed. We hypothesize that a germline-targeting immunogen capable of priming V2-apex precursor B cells in humans will need to have substantial affinity for diverse PCT64 and/or PG9/16 precursors with diverse HCDR3 V-D and D-J junctions found in human naive and/or memory B cells, such as those shown in Table S5. However, we have not detected binding of our current ApexGT trimers to such diverse precursors. Therefore, the next step toward the goal of developing a priming immunogen for humans is to develop even more advanced ApexGT trimers with affinity for diverse human precursors. The four trimer-Fab complex structures reported here were determined at different times during the study and hence were not all available to guide the design of our current most advanced immunogen, ApexGT5; further design improvements can be made by accounting for all four trimer-Fab complex structures. For immunogen evaluation, this study reports biophysical and structural analyses but does not report immunization testing; a companion paper (Melzi et al., 2022) reports immunization testing. The analyses in this manuscript do not address the potential effects of non-bnAb-precursor competitor B cells specific for the V2-apex epitope or for other epitopes on an ApexGT trimer. Our companion manuscript (Melzi et al., 2022) reports immunization studies with low bnAb-precursor B cell frequencies that allow for competition from wild-type mouse B cells. Future pre-clinical studies should include human naive B cell sorting with ApexGT trimers as bait to evaluate the human repertoire of ApexGT trimer-binding bnAb-precursors and competitors (Havenar-Daughton et al., 2018; Jardine, 2016; Steichen et al., 2019). Future pre-clinical studies should also include immunization experiments in animal models that can produce long-HCDR3 B cells for both bnAb precursors and competitors, as humans can, to enable testing the effect of competitors that have the structural capacity to reach past the glycan shield and engage protein components of the V2-apex epitope.

## STAR★Methods

### Key resources table

**Table:** 

REAGENT or RESOURCE	SOURCE	IDENTIFIER
**Antibodies**		

4025	Gorny et al., 2011	N/A
B6	Burton et al., 1991	N/A
F105	Posner et al., 1991	N/A
PGT151	Falkowska et al., 2014	N/A
PGT121	Walker et al., 2011	RRID: AB_2491041
12A12	Scheid et al., 2011	RRID: AB_2491036
PGT145	Walker et al., 2011	RRID: AB_2491054
PG9	Walker et al., 2009	RRID: AB_2491030
PCT64.35S	Landais et al., 2017	N/A
PCT64.35K	Landais et al., 2017	N/A
PCT64.LMCA	Landais et al., 2017	N/A
PCT64.iGL	Landais et al., 2017	N/A
PCT64.LMCA.JRev	This study	N/A
PG9.iGL	This study	N/A

**Bacterial and virus strains**		

Electromax Stb4	ThermoFisher	Cat# 11635018
DH5a	ThermoFisher	Cat# 18265017

**Chemicals, peptides, and recombinant proteins**		

Microcon Ultracel PL-10 centrifugal filters	Millipore Sigma	Cat# MRCPRT010
Pierce TCEP HCl	Thermo Scientific	Cat# 20490
Ammonium acetate ≥98%	Sigma-Aldrich	Cat# A1542
2-Chloroacetamide ≥98%	Sigma-Aldrich	Cat# 22790
Endo H	NEB	Cat# P0702
Calcium chloride	Sigma-Aldrich	Cat# C5670
Proteinase K	Sigma-Aldrich	Cat# P2308
PNGase F	NEB	Cat# P0705
Ammonium bicarbonate ≥99.5%	Sigma Aldrich	Cat# 09830
H218O (97% 18O)	Sigma-Aldrich	Cat# 329878
Resin, Acquity UPLC® BEH 1.7 μm C18	Waters	Cat# 186004690
Water (Optima LC-MS grade)	Fisher Scientific	Cat# W6-4
Acetonitrile	Honeywell	Cat# AH015-4
Formic acid 90%	J.T.Baker	Cat# 0129-01
Polymicro fused silica capillary tubing 100 μm	Molex	Cat# 1068150023
11 PP Vial Crimp/Snap 250 μL	Thermo Scientific	Cat# C4011-13
Snap-it Seal	Thermo Scientific	Cat# C4011-53
0.2 mL Thin-walled Tubes	Thermo Scientific	Cat# AB-0620
Protein LoBind Tube 1.5 mL	Eppendorf	Cat# 022431081
DeckWorks low binding pipette tips	Corning	Cat# 4151
rProteinA Fast Flow	GE	Cat# 17-1279-03
IgG Elution Buffer	Thermo Scientific	Cat# 210099
Pierce HeLa protein digest standard	Thermo Scientific	Cat# 88329
Opti-MEM	Thermo Scientific	Cat# 31985-088
PEI Max	FisherScientific	Cat# NC1038561
Gibson Assembly Master Mix	NEB	Cat# E2611L
LR Clonase II	ThermoFisher	Cat# 11791100
Puromycin	ThermoFisher	Cat# A1113803
Blasticidin	ThermoFisher	Cat# A1113903
Advanced DMEM	Gibco	Cat# 12491015
Tet Approved FCS	Gibco	Cat# A4736301
2-mercaptoethanol	Gibco	Cat# 21985023
Antibiotic-Antimycotic	Gibco	Cat# 15240096
Fugene HD	Promega	Cat# 32043
FITC-labeled α-cMyc	Immunolgy Consultants Lab	Cat# CMYC-45F
APC Anti-Human IgG	Jackson	Cat# 709-136-149
PE anti-V5 Tag	ThermoFisher	Cat# 12-6796-42
6x-His Epitope Tag Antibody	GenScript	Cat# 10500-530
HBS-EP+ 20x, pH 7.6	TekNova	Cat# H8022
Human Antibody Capture Kit	GE	Cat# BR-1008-39
Peroxidase AffiniPure Goat Anti-Human IgG, Fcγ	Jackson	Cat# NC9752299
Acetonitrile, 80%, 20%	Fisher Scientific	Cat# 15431423
Water with 0.1% Formic Acid	Fisher Scientific	Cat# LS118-212
Acetonitrile	Fisher Scientific	Cat# 10489553
Trifluoroacetic acid	Fisher Scientific	Cat# 10155347
Procainamide hydrochloride	Abcam	Cat# ab120955
Dithiothreitol	Sigma-Aldrich	Cat# 43819
Iodacetamide	Sigma-Aldrich	Cat# I1149
Sequencing grade chymotrypsin	Promega	Cat# V1061
Alpha Lytic protease	Sigma Aldrich	Cat# A6362
Mass spectrometry grade trypsin	Promega	Cat# V5280
Lauryl maltose neopentyl glycol	Anatrace	Cat# NG310 1 GM
293Fectin	ThermoFisher	Cat# 12347500
TransIT-mRNA transfection Kit	Mirus	Cat# MIR 2250
Alexa Fluor 647-conjugated anti-human IgG	Jackson	Cat# 109-605-003
30KD Amicon Ultra	Millipore	Cat# Z717185
1.2/1.3 C-Flat holey carbon grids	Electron Microscopy Sciences	Cat# CDFT823-50

**Deposited data**		

Mass spectrometry and proteomics analysis	Pride	Pride: PXD033766
Raw NGS Sequences	Briney et al., 2019	SRA: SRP172564
Processed NGS Dataset	(Steichen et al., 2019); https://github.com/SchiefLab/SteichenScience2019	N/A
ApexGT2.2MUT + PCT64 LMCA Fab	RCSB	PDB: 7T73
ApexGT2 + PCT64.35S Fab + RM20A3 Fab	RCSB	PDB: 7T74
ApexGT2 + RM20A3 Fab	RCSB	PDB: 7T75
ApexGT3 + PG9 iGL Fab	RCSB	PDB: 7T76
ApexGT3.2MUT + PG9 Fab	RCSB	PDB: 7T77
ApexGT2.2MUT + PCT64 LMCA Fab	EMDB	EMDB: 25732
ApexGT2 + PCT64.35S Fab+ RM20A3 Fab	EMDB	EMDB: 25733
ApexGT2 + RM20A3 Fab	EMDB	EMDB: 25734
ApexGT3 + PG9 iGL Fab	EMDB	EMDB: 25735
ApexGT3.2MUT + PG9 Fab	EMDB	EMDB: 25736
PCT64.LMCA Fab	RCSB	PDB: 7TI6

**Cell lines**		

HEK 293F	ThermoFisher	Cat# R79007; RRID: CVCL_6642

**Experimental models: Cell lines**		

rtTA3G 293T	David Nemazee, Scripps Researsh, La Jolla (Ota et al., 2012)	N/A

**Oligonucleotides**		

Loop2b OligoLibrary	CustomArray	N/A
NNK Library 32-185H	SGI-DNA	N/A
Long Ultramers NNK	IDT	N/A

**Recombinant DNA**		

pHL-SEC	Aricescu et al., 2006	Addgene #99845
pFUSE2ss-CHIg-hG1	InvivoGen	Cat# pfusess-hchg1
pFUSE2-CLIg-hL2	InvivoGen	Cat# pfuse2-hcll2
pFUSE2-CLIg-hK	InvivoGen	Cat# pfusess-hchg1
pENTR/D-TOPO	Ota et al., 2012	N/A
pLenti-CMVTRE3G/Puro_Dest	Ota et al., 2012	N/A
psPAX2	Didier Trono, EPFL, Lausanne Switzerland	AddGene#12260
pMD2.G	Didier Trono, EPFL, Lausanne Switzerland	AddGene#12259

**Software and algorithms**		

ProLuCID	http://fields.scripps.edu/yates/wp/?page_id=821	N/A
DTASelect2.0	http://fields.scripps.edu/yates/wp/?page_id=816	N/A
Census2.0	http://fields.scripps.edu/yates/wp/?page_id=824	N/A
GlycoMSQuant v.1.8.2	https://github.com/proteomicsyates/GlycoMSQuant	N/A
RAWConverter	http://fields.scripps.edu/rawconv/	N/A
Office 365	https://www.office.com/	N/A
XCalibur	Thermo Scientific	Cat# OPTON-30965-7
Abstar	(Briney et al., 2019); https://github.com/briney/abstar	N/A
Amazon EMR 6.4.0	https://aws.amazon.com/emr/	N/A
Rosetta	https://www.rosettacommons.org/software/license-and-download	N/A
ProteON Manager Software	Bio-RAD	N/A
ByosTM (Version 3.9)	Protein Metrics Inc.	N/A
GraphPad Prism v9	GraphPad	N/A
XCalibur Version v4.2	Thermo Fisher	N/A
Orbitrap Fusion Tune application v3.2	Thermo Fisher	N/A
MotionCor2	Zheng et al., 2017	N/A
Relion-2/3	Kimanius et al., 2016; Zivanov et al., 2018	N/A
SWISS-MODEL	Waterhouse et al., 2018	N/A
SabPred	Dunbar et al., 2016	N/A
UCSF Chimera	Pettersen et al., 2004	N/A
GCTF	Zhang, 2016	N/A
COOT	Emsley and Cowtan, 2004	N/A
MolProbity	Chen et al., 2010	N/A
EMRinger	Barad et al., 2015	N/A
Privateer	Agirre et al., 2015	N/A
Segger	Pintilie and Chiu, 2012	N/A
Illustrator	Adobe	N/A
**Leginon**	Suloway et al., 2005	N/A
BD FACS Diva 6	BD	N/A
FlowJo v10.8	BD	N/A

**Other**		

Influx	BD	N/A
ProteOn XPR36	Bio-Rad	Cat# 176-0100
ProteOn GLC Sensor Chip	Bio-Rad	Cat# 76-5011
MicroCal VP-Capillary DSC	Malven	N/A
Dawn HELEOS II	Wyatt	N/A
Optilab T-REX	Wyatt	N/A
GenWiz EZ Amplicon Service	Genewiz/Azenta	N/A
HisTrap HP	Cytiva/GE	Cat# 17371205
Superdex 200 10/300 GL	Cytiva/GE	Cat# 17517501
EasySpray PepMap RSLC C18 column	Thermo Fisher Scientific	Cat# ES905
PVDF protein-binding membrane	Millipore	Cat# MAIPS4510
C18 ZipTip	Merck Milipore	Cat# ZTC18S008
Vivaspin 500, 3 kDa MWCO, Polyethersulfone	Sigma-Aldrich	Cat# GE28-9322-18
PepMap100 C18 3UM 75UMx2CM Nanoviper	Thermo Scientific	Cat# 164946
Vitrobot mark IV	Thermo Fisher Scientific	N/A
Titan Krios	Thermo Fisher Scientific	N/A
Talos Arctica	Thermo Fisher Scientific	N/A
K2 Summit direct electron detector	Gatan	N/A
Unformulated mRNA	Moderna	N/A
NovoCyte 3000	Agilent	N/A

### Resource availability

#### Lead contact

Further information and requests for resources and reagents should be directed to and will be fulfilled if practicable by the lead contact, William R. Schief (schief@scripps.edu).

#### Materials availability

Plasmids generated in this study will be made available under Uniform Biological Material Transfer Agreement (UBMTA) from The Scripps Research Institute.

### Experimental model and subject details

#### Cell lines

This study used HEK 293F cells, for production of soluble proteins in suspension and for cell-surface expression and antigenicity testing, and rtTA3G-expressing HEK 293T cells, for mammalian display directed evolution. 293T cells were cultured at 37°C in Advanced DMEM (Gibco) supplemented with 5% FCS, GlutaMAX (Gibco), 2-mercaptoethanol (Gibco) and Antibiotic-Antimycotic (Gibco); rtTA3G-expressing HEK 293T cells were cultured under the same conditions but with the addition of 10 μg/mL blasticidin (Gibco). Sex of both cell lines was female. Cell lines have not been authenticated.

#### NGS dataset of human BCR HCs

This work utilized a large NGS dataset of 1.1×10^9^ amino acid sequences of BCR HCs from 14 healthy, HIV-uninfected donors as described previously (Steichen et al., 2019). Briefly, this dataset contains 255 million sequences from 10 donors obtained from VDJ heavy chain mRNA transcripts that were amplified with unique identifiers to minimize PCR bias and allow for error correction (Briney et al., 2019). In addition, 4 donors were sequenced from FW3 to FW4 using the NextSeq or HiSeq (2 x 150 bp) platform to obtain an additional 845 million sequences. Sequences were annotated with Abstar, converted to parquet format, and uploaded to AWS S3 storage platform (see https://github.com/schieflab/Willis2022. Multiple biological and technical replicates were recorded in the dataset as described previously (Briney et al., 2019).

#### Apex bnAb precursor frequency estimates

The NGS dataset was interrogated with the Spark analytics engine on AWS EMR platform (EMR 6.4.0) using the precursor definitions defined in Table S2. PySpark scripts used in this analysis are available at https://github.com/SchiefLab/Willis2022 along with instructions on setting up an EMR cluster. An EMR cluster is an AWS service that installs software on one or more main nodes along with one or more worker nodes. We used Spark, JupyterEnterpriseGateway, Hadoop and JupyterHub which was installed on all nodes. The notebook interface on EMR was used to interrogate the precursor frequencies using PySpark scripts. A precursor frequency was estimated by taking the total amount of precursors for each technical replicate that met the definitions in Table S2 divided by the total number of sequences in each replicate. The frequency per donor was averaged for each donor and the total frequency for each Apex bnAb precursor was reported as the median across all 14 donors for at least one precursor was found. To determine a precursor response, we computed the frequency of donors which at least one precursor was found. 95% confidence intervals were estimated using the Wilson method for binomial proportions (Agresti and Coull, 1998).

#### Apex bnAb precursor frequency similarity

To calculate the similarity for each bnAb Apex class, we iterated through each precursor found in our NGS query and determined the closest bnAb for that class when more than one bnAb is available, (ex. PG9 or PG16). For the closest bnAb, we aligned to the precursor and computed the edit distance, which is quantifies the similarity of two sequences of the same length. An edit distance of 2 would mean that 2 mutations are needed for the sequences to be the same. The edit distance was normalized by length of the sequence to make each bnAb class comparable. For all precursors found per donor, the mean edit distance was recorded.

#### Updating PG9 iGL

To determine the most appropriate amino acid at position 100p (kabat numbering) in PG9 iGL, we searched long HCDR3s with the following regular expression “.^∗^YYDF[WY][SD]GYY.YYYMDV$”. This regular expression queried our NGS dataset for the DJ recombination used by PG9 while leaving the ambiguous position as a “wild card” a ‘.’ In this expression. The most frequent amino acid used at the “wild card” position was then used in our definition of PG9.iGL at position. 100p.

#### DNA gene synthesis and cloning

Five types of gene constructs were used in this study. Recombinant trimers and antibodies were synthesized at Genscript, Inc. Trimers were cloned into pHLsec between the signal sequence and a C-terminal GTKHHHHHH tag using the AgeI and KpnI cloning sites. Antibody heavy chains were cloned into pFUSEss-CLIg-hL2 between the leader and IgG1 human constant domain using EcoRI and NdeI cloning sites. Kappa chains were cloned into pFUSEss-CLIg-hK between the leader and human kappa constant region using the EcoRI and BsiWI. Lambda chains were cloned into pFUSEss-CLIg-hL2 between the leader sequence and the last amino acid in lambda FW4 using the EcoRI and AvrII cloning sites.

Genes used for library design were cloned via Gibson assembly into pENTR which contained a C-terminal cMyc epitope followed by a platelet-derived growth factor receptor (PDGFR) transmembrane domain. NNK scanning libraries were prepared by SGI. NNK combinatorial libraries were ordered as ultramers from long ultramers (IDT) using ambiguous nucleotides at the combinatorial positions. Altering compliment and reverse compliment ultramers were assembled with outer primers which overlapped an insertion site by 40 bp. Gibson assembly master mix was used according to the manufacturer’s instructions but scaled by ten times to get maximum library depth.

For the Loop2b library, CustomArray oligos were amplified with insertion primers flanking the Loop2b region. Gibson assembly master mix was used according to manufactures instructions and scaled by ten times.

#### ApexGT protein expression

BG505 based ApexGT variants cloned into pHLSEC as described above and transformed into DH5a cells using the manufactures protocol. Cells were maxi-prepped with a BenchPro 2100 (Thermo Fisher Scientific). The purified constructs were transfected by incubating 300 uL PEI max with 70 ug DNA and 25 ug Furin protease encoded DNA into 15 mL OptiMEM. After 30 min, the mixture was added to 293F cells grown in 293 Freestyle media at a density of 1 million cells/mL. After 6 days, the supernatant was clarified in a centrifuge at 1000 rpm. The was purified from the supernatant using a HIS-TRAP column, starting with a wash buffer (20 mM Imidazole, 500 mM NaCl, 20 mM Na2HPO4) and mixing with elution buffer (500 mM Imidazole, 500 mM NaCl, 20 mM Na2HPO4) using a linear gradient. The trimer fraction was collected and further purified on an S200Increase 10-300 column (GE) in HEPES buffered saline (HBS) (10 mM HEPES, 150 mM NaCl). The oligomeric state of the SOSIP trimers were then confirmed by size exclusion chromatography-multi-angle light scattering using the DAWN HELEOS II multi-angle light scattering system with Optilab T-rEX refractometer (Wyatt Labs). The trimers were frozen in thin-walled PCR tubes at 1 mg/mL using liquid nitrogen and stored at −80°C.

#### Antibody protein expression

Plasmids were maxi-prepped using the BenchPro 2100 system (Thermo Fisher Scientific). Heavy and light chains were transfected with PEImax (Polysciences) in OptiMEM (Thermo Fisher Scientific) at a 3:1:1 PEI:heavy:light ratio into Freestyle 293F cells (Thermo Fisher Scientific). Supernatants were harvested on day 7, clarified by centrifugation and filtration, and purified by rProtein A Sepharose fast flow (Cytiva). After elution with IgG elution buffer (Thermo Fisher Scientific), antibodies were buffer-exchanged into Tris buffered saline (TBS).

#### ApexGT immunogen design

Lentiviral mammalian display and directed evolution were performed as described previously (Steichen et al., 2016) with modifications to sorting probes and libraries. Starting with BG505.SOSIP.D664 based construct, two library pathways were constructed. The first library used NNK degenerate codons at positions 32-185H (HXBC2 numbering, SGI). The second library had combinatorial 4 combinatorial NNK positions K170-V173 using IDT. Both libraries were subsequently cloned into and the gateway entry vector pENTR/D-TOPO (Ota et al., 2012) using the Gibson Assembly Mix (NEB) (See DNA synthesis and cloning). The purified pENTR vector was then Gateway cloned to pLenti CMVTRE3G puro Dest (Ota et al., 2012) using the LR Clonase II enzyme mix. This plasmid DNA was purified and ready for use in transfection. 293T cells cultured at 37°C in Advanced DMEM (Gibco) supplemented with 5% FCS, GlutaMAX (Gibco), 2-mercaptoethanol (Gibco) and Antibiotic-Antimycotic (Gibco) were co-transfected with NNK library in pLenti CMVTRE3G pDEST Puro (10.8 μg), psPAX2 (7.0 μg) and pMD2.G (3.8 μg) with FugeneHD in a T-75 flask (Salmon and Trono, 2007). 293T cells stably expressing rtTA3G from the pLenti CMV rtTA3G Blast vector (Ota et al., 2012) were transduced at low MOI (<0.1) in a T-75 or T-225 flask in the presence of 10 μg/mL blasticidin (Gibco). The next day cells were selected with 2 μg/mL puromycin. 293T cells containing the stable library were induced with doxycycline (1 μg/mL) and the following day were harvested in FACS buffer (HBSS, 1 mM EDTA, 0.5% BSA). Cells were incubated with PG9 VHVL washed with FACS buffer, and then stained with fluorescein isothiocyanate (FITC)-labeled α-cMyc (Immunology Consultants Laboratory) and phycoerythrin APC-conjugated αnti-human IgG (Jackson). Cells were sorted on a BD Influx (BD Biosciences) FACS sorter. Approximately 10K of the desired gates cells were collected and expanded for ∼ one week in the presence of puromycin and blasticidin before the next round of enrichment was carried out.

For the Loop2b library (D180 – Y191) we considered all natural sequences gathered from the LANL HIV Sequence Database (http://www.hiv.lanl.gov/) using semiconductor oligonucleotide arrays (CustomArray) and cloned into BG505 SOSIP.D664 to generate a library that could be sorted by mammalian display as described above but with PG9 iGL +1 MUT as the desired gate and 4025/V5 tag as negative gate to enrich for well formed “closed” trimers. The genomic DNA for the pre-sorted libraries, intermediate sorted rounds, and the final enriched libraries were extracted and PCR amplified using partial adapters recommended by GeneWiz “EZ amplicon”. We determined enriched mutations as described previously (Kulp et al., 2017; Steichen et al., 2016).

#### Surface plasmon resonance (SPR)

Kinetics and affinities of antibody-antigen interactions were measured on a ProteOn XPR36 using GLC Sensor Chip and 1x HBS-EP + pH 7.4 running buffer supplemented with BSA at 1 mg/ml. Human Antibody Capture Kit was used according to manufacturer’s instructions to immobilize about 6000 RUs of capture mAb onto each flow cell. In a typical experiment, approximately 300-400 RUs of mAbs were captured onto each flow cell and analytes were passed over the flow cell at 30 μL/min for 3 min followed by a 10 min dissociation time. Regeneration was accomplished using 3M Magnesium Chloride with 180 seconds contact time and injected four times per cycle. Raw sensograms were analyzed using ProteOn Manager software (Bio-Rad), including interspot and column double referencing, and either Equilibrium fits or Kinetic fits with Langmuir model with 1:1 binding stoichiometry, or both, were employed when applicable. Only data sets with Rmax-Ratio between 0.5 and 2 were accepted as correct. Rmax-Ratio was calculated by dividing theoretical Rmax-Expected by Rmax-Fit obtained from fitting the data. Rmax-Expected was calculated from ligand capture level assuming 2 binding sites per mAb-ligand molecule and one binding site per trimer-analyte molecule. For example, if we capture 100 RU of mAb and trimer molecular weight is 225 kDa the Rmax-Expected would be 225^∗^2^∗^100/150 = 300 RU. Analyte concentrations were measured on a NanoDrop 2000c Spectrophotometer using Absorption signal at 280 nm.

#### Differential scanning calorimetry (DSC)

DSC experiments were performed on a MicroCal VP-Capillary differential scanning calorimeter (Malvern Instruments). The HBS buffer was used for baseline scans and the protein samples were diluted into HBS buffer to adjust to 0.25 mg/mL. The system was allowed to equilibrate at 20°C for 15 min and then heat up till 90°C at a scan rate of 90°C/h. Buffer correction, normalization, and baseline subtraction were applied during data analysis using Origin 7.0 software. The non-two-state model was used for data fitting.

#### Antigenic profile with enzyme-linked immunosorbent assay (ELISA)

96-well plates were coated overnight at 4C with 6x-His Epitope Tag Antibody at 2 mg/mL in PBS. Plates were washed 3 times with PBS, 0.05% Tween (PBS-T), and blocked with 10% milk PBS for 1h. Subsequently, 2 mg/mL of the purified His-tagged ApexGT or MD39 trimers was added for 2 h in 1% milk PBS-T, after which the plates were washed three times with PBS-T. Serial dilutions of antigenic profiling mAbs PGT151, PG145, F105, PGT121, 12A12, 4025, B6, PCT64.35S and PG9 in 1% milk PBS-T were added to the plates for 1 h, after which the plates were washed again three times with PBS-T before the addition of anti-human conjugated peroxidase at 1:1000 for 1 h. After four final washes, binding was detected by the addition of TMB substrate and measured by absorbance at 405 nm.

#### Cryo-EM sample preparation

Purified ApexGT2.2MUT, Apex3, or ApexGT3.N130 was incubated overnight at 4° with ∼6 molar excess purified PCT64.LMCA, PCT64.35S, PG9.iGL, PG9, and/or RM20A3 Fab then purified via size exclusion chromatography on a Superdex 200 Increase column followed by concentration of pooled fractions with a 30kD molecular weight cut-off using an Amicon Ultra centrifugal filter to a final concentration of ∼3-7 mg/ml. Concentrated sample was mixed with 0.5μL of 0.04mM lauryl maltose neopentyl glycol (LMNG; Anatrace) to a final concentration of 0.005 mM and 4 μL of this solution was applied to plasma cleaned 1.2/1.3 C-Flat holey carbon grids (Electron Microscopy Sciences) using a Vitrobot mark IV (Thermo Fisher Scientific) with a 7sec blot time, 0 blot force, and wait time of 0 sec. Prepared grids were then stored in liquid nitrogen until they were transfer to a microscope for imaging.

#### Cryo-EM data collection

A table of detailed imaging conditions and data statistics for all the EM datasets is presented in Supplemental Table 6. All datasets were collected with Legion automated microscopy software (Suloway et al., 2005) on either an FEI Titan Krios operating at 300keV or an FEI Talos Arctica operating at 200keV Thermo Fisher Scientific), both equipped with a K2 Summit direct electron detector (Gatan) operated in counting mode.

#### Cryo-EM data processing

All movie micrographs were aligned and dose-weighted using MotionCor2 (Zheng et al., 2017) and CTF parameters were estimated with GCTF (Zhang, 2016) Single-particle processing was carried out using a combination of either Relion-2/3 (Kimanius et al., 2016; Zivanov et al., 2018) and CryoSparc2 (Punjani and Fleet, 2021). The ApexGT2.2MUT + PCT64.LMCA, and both ApexGT3 datasets were collected several years earlier than the ApexGT2.2MUT + PCT64.35S dataset and were processed initially using exclusively Relion2. However, because improved software became available during the preparation of this paper, particles from each dataset were uploaded to CryoSparc2 and further processed as shown in Figures S11, S12, S16 and S17. The ApexGT3A + PG9.iGL and ApexGT3A.N130 + PG9 complexes were both imaged in two sessions on different microscopes and combined by down sampling the higher magnification data to match the final pixel size of the lower magnification data using Relion-2. The following general workflow was used for all datasets presented in this study. After frame alignment, dose-weighting, and CTF estimation, micrographs were sorted based on CTF fit parameters and particle picking was performed first using a gaussian blot template on a subset of micrographs. These particles were then extracted, aligned and classified in 2-D, and the class averages were then used for template picking of the full dataset. Picked particles were extracted and subjected to one or two rounds of 2D-classificaiton followed by subset selection and re-extraction with re-centering. One round of ab initio classification was carried out followed by subset selection of all classes containing well refined trimers. After subset selection, 3D-autorefinement was performed with per-particle CTF estimation followed by another round of 3-D classification, this time using the newly developed 3-D variability algorithm implemented in CryoSparc2. A soft spherical mask that surrounds the trimer apex and large enough to accommodate the entire Fab was used to isolate variability in Fab occupancy followed by clustering into 3-6 classes. Clusters with clear density for Fab were then pooled and refined again together. 3-D variability was then employed again for both PCT64 datasets, this time to isolate variability in Fab binding angle followed by clustering and pooling of particles with similar angle of approach. Lastly, a single final round of 3-D non-uniform refinement with per-particle CTF estimation and correction were performed to generate the published reconstructions. For the apo ApexGT2.2MUT structure, C3 symmetry was imposed during refinement.

#### Model building and figure preparation

Model building was initiated by preparing a monomeric Env homology model with SWISS-MODEL (Waterhouse et al., 2018) using MD39-10MUTA (PDB: 5T3S) as a template. A homology model of PG9.iGL was generated using SAbPred (Dunbar et al., 2016), while crystal structures were used for PG9 (PDB:3U4E), PCT64.LMCA (PDB:6CA9), and PCT64.35S (PDB:6CA6). Preliminary Env and Fab models were then fit into cryo-EM maps and combined into a single PDB file using UCSF Chimera (Pettersen et al., 2004). Initial refinement was done using Rosetta (Wang et al., 2016). Glycans were then added manually and refined using COOT (Wang et al., 2016). Fully glycosylated models were then refined again in Rosetta asking for ∼300 models. All models were validated using MolProbity (Chen et al., 2010) and EMRinger (Barad et al., 2015) and the model with the lowest combined score was selected. All models were then checked and adjusted manually in COOT and re-refined with Rosetta, if necessary, then renumbered to match Kabat and HXB2 numbering schemes in the python scripting interface of COOT. Final models were then scored again with MolProbity and EMRinger, while glycan structures were further validated with Privateer (Agirre et al., 2015). Figures were prepared with either UCSF Chimera or ChimeraX (Pettersen et al., 2021). Electrostatic potential surfaces were calculated according to Coulomb’s law and visualized using Chimera. Hydrogen bonds were calculated and displayed with UCSF ChimeraX. Volume segmentation was performed with Segger (Pintilie et al., 2010) as implemented in UCSF ChimeraX. Interface surface area was calculated using PDBePISA (Krissinel and Henrick, 2007). Figures were prepared in Adobe Illustrator (Adobe Inc.) and PowerPoint (Microsoft) and Supplemental movies were edited with Blender.

#### Glycan analysis

Two methods were used in glycan analysis.

Method 1: DeGlyPHER (Baboo et al., 2021) was used to ascertain site-specific glycan occupancy and processivity on the examined glycoproteins.

#### Glycan analysis proteinase K treatment and deglycosylation

HIV Env glycoprotein was exchanged to water using Microcon Ultracel PL-10 centrifugal filter. Glycoprotein was reduced with 5 mM tris(2-carboxyethyl)phosphine hydrochloride (TCEP-HCl) and alkylated with 10 mM 2-Chloroacetamide in 100 mM ammonium acetate for 20 min at room temperature (RT, 24ᵒC). Initial protein-level deglycosylation was performed using 250 U of Endo H for 5 μg trimer, for 1 h at 37ᵒC. Glycorotein was digested with 1:25 Proteinase K (PK) for 30 min at 37ᵒC. PK was denatured by incubating at 90ᵒC for 15 min, then cooled to RT. Peptides were deglycosylated again with 250 U Endo H for 1 h at 37ᵒC, then frozen at −80ᵒC and lyophilized. 100 U PNGase F was lyophilized, resuspended in 20 μL 100 mM ammonium bicarbonate prepared in H218O, and added to the lyophilized peptides. Reactions were then incubated for 1 h at 37ᵒC, subsequently analyzed by LC-MS/MS.

#### Glycan analysis LC-MS/MS

Samples were analyzed on an Q Exactive HF-X mass spectrometer. Samples were injected directly onto a 25 cm, 100 μm ID column packed with BEH 1.7 μm C18 resin. Samples were separated at a flow rate of 300 nL/min on an EASY-nLC 1200 UHPLC. Buffers A and B were 0.1% formic acid in 5% and 80% acetonitrile, respectively. The following gradient was used: 1–25% B over 160 min, an increase to 40% B over 40 min, an increase to 90% B over another 10 and 30 min at 90% B for a total run time of 240 min. Column was re-equilibrated with solution A prior to the injection of sample. Peptides were eluted from the tip of the column and nanosprayed directly into the mass spectrometer by application of 2.8 kV at the back of the column. The mass spectrometer was operated in a data dependent mode. Full MS1 scans were collected in the Orbitrap at 120,000 resolution. The ten most abundant ions per scan were selected for HCD MS/MS at 25 NCE. Dynamic exclusion was enabled with exclusion duration of 10 s and singly charged ions were excluded.

#### Glycan analysis data processing

Protein and peptide identification were done with Integrated Proteomics Pipeline (IP2). Tandem mass spectra were extracted from raw files using RawConverter (He et al., 2015) and searched with ProLuCID (Xu et al., 2015) against a database comprising UniProt reviewed (Swiss-Prot) proteome for *Homo sapiens* (UP000005640), UniProt amino acid sequences for Endo H (P04067), PNGase F (Q9XBM8), and PK (P06873), amino acid sequences for the examined proteins, and a list of general protein contaminants. The search space included no cleavage-specificity. Carbamidomethylation (+57.02146C) was considered a static modification. Deamidation in presence of H218O (+2.988261 N), GlcNAc (+203.079373 N), oxidation (+15.994915 M) and N-terminal pyroglutamate formation (−17.026549 Q) were considered differential modifications. Data was searched with 50 ppm precursor ion tolerance and 50 ppm fragment ion tolerance. Identified proteins were filtered using DTASelect2 (Tabb et al., 2002) and utilizing a target-decoy database search strategy to limit the false discovery rate (FDR) to 1%, at the spectrum level (Peng et al., 2003). A minimum of 1 peptide per protein and no tryptic end per peptide were required and precursor delta mass cut-off was fixed at 15 ppm. Statistical models for peptide mass modification (modstat) were applied. Census2 (Park et al., 2008) label-free analysis was performed based on the precursor peak area, with a 15 ppm precursor mass tolerance and 0.1 min retention time tolerance. “Match between runs” was used to find missing peptides between runs. Data analysis using GlycoMSQuant (Baboo et al., 2021) was implemented to automate the analysis. GlycoMSQuant summed precursor peak areas across replicates, discarded peptides without NGS, discarded misidentified peptides when N-glycan remnant-mass modifications were localized to non-NGS asparagines and corrected/fixed N-glycan mislocalization where appropriate. The results were aligned to NGS in Env of HXB2 (Tian et al., 2016) HIV-1 variant.

Method 2: Single site glycan profiling (Allen et al., 2021) was used for ApexGT2 glycan analysis.

Three aliquots were denatured for 1h in 50 mM Tris/HCl, pH 8.0 containing 6 M of urea and 5 mM dithiothreitol (DTT). Next, Env proteins were reduced and alkylated by adding 20 mM iodoacetamide (IAA) and incubated for 1h in the dark, followed by a 1h incubation with 20 mM DTT to eliminate residual IAA. The alkylated Env proteins were buffer-exchanged into 50 mM Tris/HCl, pH 8.0 using Vivaspin columns (3 kDa) and two of the aliquots were digested separately overnight using trypsin, chymotrypsin (Mass Spectrometry Grade, Promega) or alpha lytic protease (Sigma Aldrich) at a ratio of 1:30 (w/w). The next day, the peptides were dried and extracted using C18 Zip-tip (MerckMilipore). The peptides were dried again, re-suspended in 0.1% formic acid and analyzed by nanoLC-ESI MS with an Ultimate 3000 HPLC (Thermo Fisher Scientific) system coupled to an Orbitrap Eclipse mass spectrometer (Thermo Fisher Scientific) using stepped higher energy collision-induced dissociation (HCD) fragmentation. Peptides were separated using an EasySpray PepMap RSLC C18 column (75 μm × 75 cm). A trapping column (PepMap 100 C18 3μM 75μM x 2cm) was used in line with the LC OBJprior to separation with the analytical column. The LC conditions were as follows: 280 min linear gradient consisting of 4-32% acetonitrile in 0.1% formic acid over 260 min followed by 20 min of alternating 76% acetonitrile in 0.1% formic acid and 4% Acn in 0.1% formic acid, used to ensure all the sample had eluted from the column. The flow rate was set to 200 nL/min. The spray voltage was set to 2.7 kV and the temperature of the heated capillary was set to 40°C. The ion transfer tube temperature was set to 275°C. The scan range was 375−1500 m/z. Stepped HCD collision energy was set to 15, 25 and 45% and the MS2 for each energy was combined. Precursor and fragment detection were performed using an Orbitrap at a resolution MS1 = 120,000. MS2 = 30,000. The AGC target for MS1 was set to standard and injection time set to auto which involves the system setting the two parameters to maximize sensitivity while maintaining cycle time. Full LC and MS methodology can be extracted from the appropriate Raw file using XCalibur FreeStyle software or upon request.

Glycopeptide fragmentation data were extracted from the raw file using Byos (Version 3.5; Protein Metrics Inc.). The glycopeptide fragmentation data were evaluated manually for each glycopeptide; the peptide was scored as true-positive when the correct b and y fragment ions were observed along with oxonium ions corresponding to the glycan identified. The MS data was searched using the Protein Metrics 305 N-glycan library with sulfated glycans added manually. The relative amounts of each glycan at each site as well as the unoccupied proportion were determined by comparing the extracted chromatographic areas for different glycotypes with an identical peptide sequence. All charge states for a single glycopeptide were summed. The precursor mass tolerance was set at 4 ppm and 10 ppm for fragments. A 1% FDR was applied. The relative amounts of each glycan at each site as well as the unoccupied proportion were determined by comparing the extracted ion chromatographic areas for different glycopeptides with an identical peptide sequence. Glycans were categorized according to the composition detected.

HexNAc(2)Hex(10+) was defined as M9Glc, HexNAc(2)Hex(9−5) was classified as M9 to M3. Any of these structures containing a fucose were categorized as FM (fucosylated mannose). HexNAc(3)Hex(5–6)X was classified as Hybrid with HexNAc(3)Hex(5-6)Fuc(1)X classified as Fhybrid. Complex-type glycans were classified according to the number of HexNAc subunits and the presence or absence of fucosylation. As this fragmentation method does not provide linkage information compositional isomers are grouped, so for example a triantennary glycan contains HexNAc 5 but so does a biantennary glycans with a bisect. Core glycans refer to truncated structures smaller than M3. M9glc- M4 were classified as oligomannose-type glycans. Glycans containing at least one sialic acid or one sulfate group were categorized as NeuAc and sulfated respectively.

#### Cell surface display antigenic profiling

Unformulated membrane bound mRNA immunogens (Moderna) were transfected into HEK293F suspension cells grown in 293 Freestyle media (Life Technologies) by the Mirus TransIT-mRNA transfection Kit (MIR 2250) and incubated at 37°C, 125rpm for 24hrs. Each antibody solution for antigenic profile test (in Figure 7C) was prepared at 10 ug/mL in FACS buffer (HBSS, 1 mM EDTA, 1% BSA). To note, trimer-specific bnAbs (interface/FP: PGT151, V2 apex: PGT145, PG9 and PCT64.35S) and non-nAbs (V3: 4025, CD4bs: B6 and F105) were selected to characterize the open vs. closed nature of the membrane bound trimer; not-trimer-specific bnAbs (N332: PGT121 and CD4bs: 12A12) were selected to evaluate cell surface immunogen expression; germline reverted variants of PG9 and PCT64 (PG9.iGL, PCT64.LMCA, PCT64.LMCA.Jrev and PCT64.iGL) were selected to assess binding capacity of cell surface immunogen towards V2 bnAbs-like precursors.

Cell suspension was distributed onto a deep-well 96-well plate at 1 mL per well and harvested at 500 g for 5 min. Each well of cells was resuspended by 100 uL of 10 ug/mL mAb solution and incubated at 37°C, 125rpm for 1 hr. Cells were washed twice with 150 uL FACS buffer and then stained with SYTOX™ Green Dead Cell Stain (Invitrogen) and Alexa Fluor 647-conjugated anti-human IgG (Jackson Immuno Research) at 37°C, 125rpm for 20 min. Cells were analyzed on a NovoCyte 3000 with NovoSampler Pro FACS sorter (Agilent ACEA) by a BD FACS Diva 6 software (BD Biosciences). Approximately 50k live cells were collected per well. Data was analyzed using FlowJo™ v10.8 Software. The methods for cell surface antigenic profiling for DNA-transfected cells were similar to those for mRNA, except that DNA-encoded membrane bound trimers were transfected by the 293fectin Transfection Reagent (Gibco) and incubated for 2 days before FACS.

### Quantification and statistical analysis

Median precursor detection rates across n = 14 donors, with 95% confidence intervals computed using the Wilson score method (Agresti and Coull, 1998), were reported in Figure 2C. Median precursor frequencies, computed over donors with non-zero frequencies (n = 14 for PCT64, n = 9 for PG9/16, n = 6 for PGT/PGDM, n = 2 for CH01-04, n = 1 for CAP256), were reported in Figure 2D. Mean HCDR3 distance to bnAb, computed over donors with at least one detectable bnAb precursor shown in Figure 2D, was reported in Figure 2E.

## Data Availability

• Processed next-generation sequencing (NGS) data used in this study can be found in parquet format at s3://steichenetalpublicdata/analyzed_sequences/parquet/ and are available as of the date of publication. A tutorial on using this format can be found at https://github.com/schieflab/Willis2022. Example queries using PySpark are also available along with instructions on setting up an Amazon Web Service (AWS) Elastic Map Reduce (EMR) Cluster. Raw NGS sequences for 10 of the 14 donors were previously deposited in the Sequence Read Archive (SRA), are available as of the date of publication (Briney et al., 2019), and have accession numbers provided in the KRT. Cryo-electron microscopy (cryo-EM) structure data for five high-resolution complexes were deposited into the RCSB Protein Data Bank (PDB) and Electron Microscopy Databank (EMDB) and are available as of the date of publication. Crystal structure data for one unliganded Fab were deposited into the PDB and are available as of the date of publication. Mass spectrometry proteomic data for glycan analysis was deposited into the PRoteomics IDEntifications (PRIDE) database and are available as of the date of publication. Accession numbers are listed in the KRT for data deposited to the PDB, EMDB, and PRIDE. • This paper does not report original code. • Any additional information required to reanalyze the data reported in this paper is available from the lead contact upon request.

## Supplemental information

Document S1. Figures S1–S7 and Table S1–S6

Document S3. Article plus supplemental information
