# Supplementary material for: Human immunoglobulin repertoire analysis guides design of vaccine priming immunogens targeting HIV V2-apex broadly neutralizing antibody precursors
Source: Immunity. 2022 Nov 8;55(11):2149–2167.e9. doi: 10.1016/j.immuni.2022.09.001 (PMC9671094; doi:10.1016/j.immuni.2022.09.001)
Supplement: Document S1. Figures S1–S7 and Table S1–S6 [file mmc1.pdf]

Immunity, Volume *55*

Supplemental information

Human immunoglobulin repertoire analysis guides design of vaccine priming immunogens targeting HIV V2-apex broadly neutralizing antibody precursors

Jordan R. Willis, Zachary T. Berndsen, Krystal M. Ma, Jon M. Steichen, Torben Schiffner, Elise Landais, Alessia Liguori, Oleksandr Kalyuzhniy, Joel D. Allen, Sabyasachi Baboo, Oluwarotimi Omorodion, Jolene K. Diedrich, Xiaozhen Hu, Erik Georgeson, Nicole Phelps, Saman Eskandarzadeh, Bettina Groschel, Michael Kubitz, Yumiko Adachi, Tina-Marie Mullin, Nushin B. Alavi, Samantha Falcone, Sunny Himansu, Andrea Carfi, Ian A. Wilson, John R. Yates III, James C. Paulson, Max Crispin, Andrew B. Ward, and William R. Schief

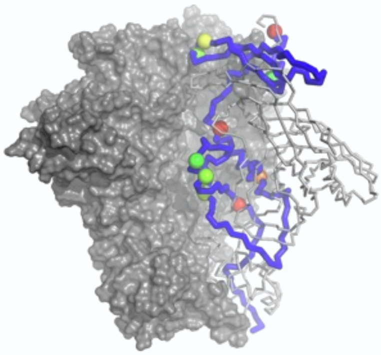

NNK Scan

Description	Sorted With
- 150 NNK positons from 32-185$_H$ -Skips cysteines	- PG16 HCDR3$_{Rev}$

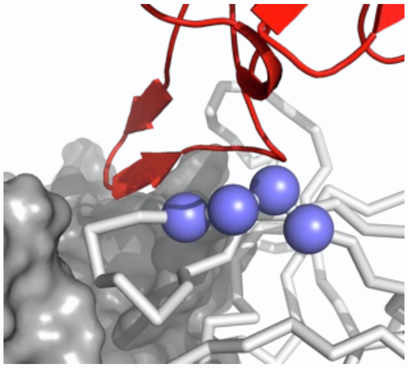

NNK Combination

Description	Sorted With
- 4 NNK Combinations at interface K170-V173	- PG9<br>- PG16<br>- PG9 HCDR3$_{Rev}$ + 1MUT<br>- PG16 $V_H/V_{L\ Rev}$

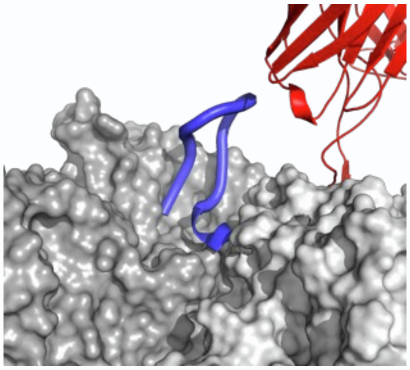

Loop2b

Description	Sorted With
- All natural loop sequences from LANL<br>- D180 – Y191	-PG9<br>-PG9 $V_H/V_{L\ Rev}$<br>-PG9 iGL + 1MUT<br>-PG9 iGL<br>-PGT145<br>- 4025 (negative sort)

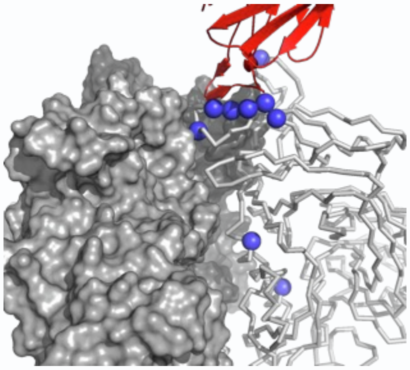

Gen 2. Combination

Description	Sorted With
- Bootstrapped positions from NNK combinations and NNK scan	-PG9<br>-PG9 HCDR3$_{Rev}$ + 1MUT<br>-PG9 $V_H/V_{L\ Rev}$<br>-PG9 iGL + 1MUT<br>-PG9 iGL<br>- 4025 (negative sort)

Figure S1. Trimer libraries and selection reagents support directed evolution of ApexGT trimers. ApexGT model shown in grey surface representation. Regions of blue are the library positions. Sequences of sorting probe are shown in Figure S2A. (related to **Fig 4**)

A

B

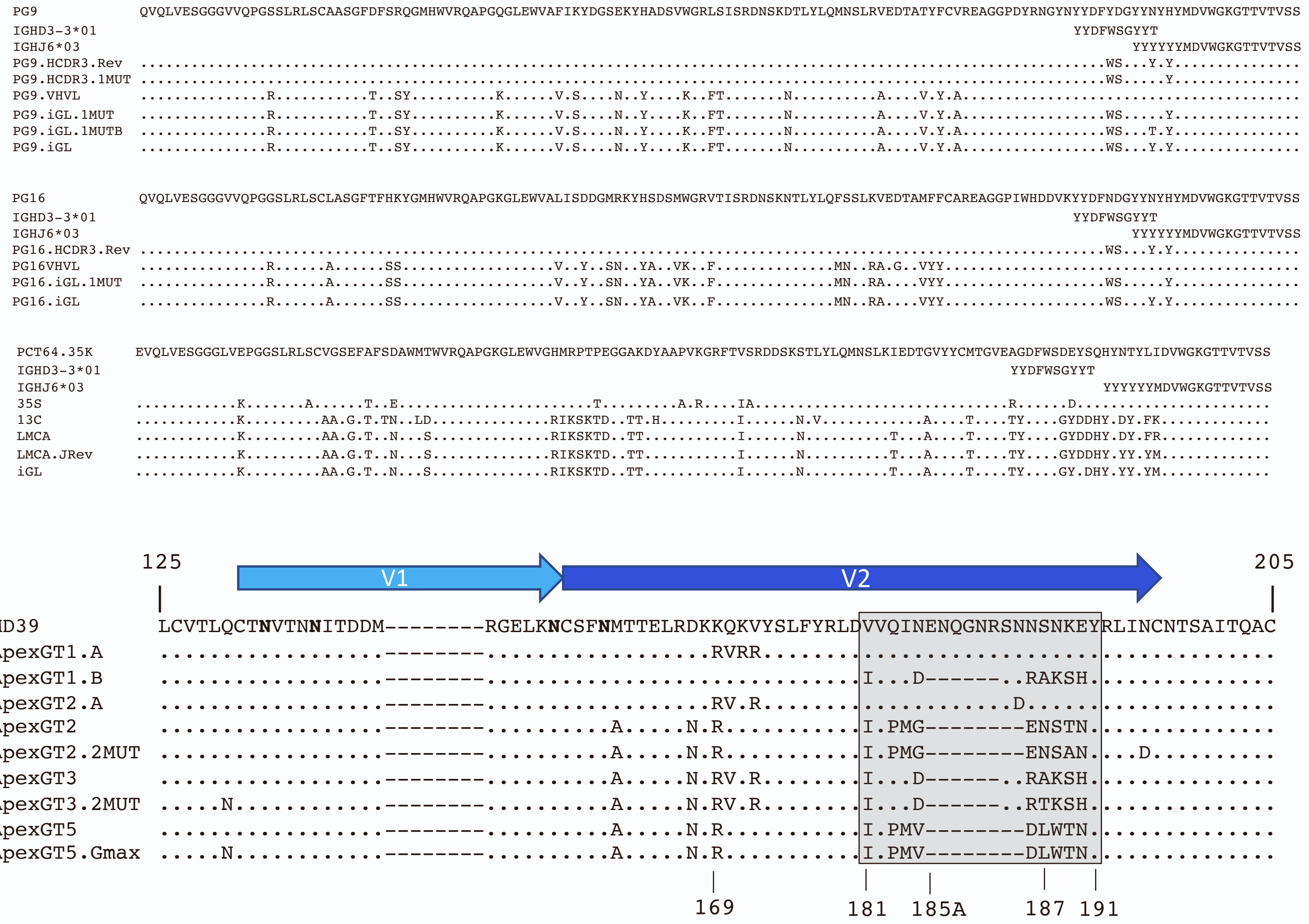

C

		BG505	MD39	ApexGT1 A	ApexGT1 B	ApexGT2 -	ApexGT2 A	ApexGT2 2MUT	ApexGT2 GMAX	ApexGT3 -	ApexGT3 GMAX	ApexGT5 -	ApexGT5 GMAX
PCT64	PCT64.35K	-	710	-	-	18	-	-	3.5	2.3	7.2	0.92	0.544
	PCT64.13C	>	>	274	4180	1.0	159	1.0	-	-	-	-	-
	PCT64.LMCA	>	>	>*	-	167	-	78	165	1110	5520	66	121
	PCT64.LMCA.J	>	>	>*	-	6400	-	-	7400	>*	>*	347	757
	PCT64.iGL	>	>*	>*	>*	>*	>*	>	>*	>*	>*	3600	4740
PG9/PG16	PG9	15.8	17.4	1.3	21.5	0.7	2.4	2.4	4.5	0.2	2.0	8.59	14.2
	PG9.VHVL	72	242	2	-	-	-	-	-	-	-	-	-
	PG9.HCDR3 + 1MUT	317	579	88	-	-	-	-	-	-	-	-	-
	PG9.iGL +1MUT	645	1420	396	625	88	49	91.9	-	4	-	-	-
	PG9.iGL	>	>*	1230	7060	1870	>*	-	3860	104	71.4	596	626
	PG16	38	32	2	-	-	-	-	-	-	-	-	-
	PG16.HCDR3 + 1MUT	273	628	172	-	-	-	2320	-	-	-	-	-
	PG16.VHVL	1001	>	18	-	-	-	-	-	-	-	-	-
	PG16.iGL	>	>	>	>	1400	-	-	-	-	-	-	-

> No binding detected at 5000 nM

>* No binding detected at 10000 nM

KD (nM)

Figure S2. ApexGT trimer alignment and extended binding analysis characterize iterative immunogen design.

ApexGT model shown in grey surface representation. Regions of blue are the library positions. Sequences of sorting probe are shown in Figure S2A. **(A)** Amino acid sequence alignments for PCT64 and PG9 variants used in this study, aligned to their inferred germline VDJ segments. **(B)** Amino acid sequences of MD39 and ApexGT variants shown for positions 125-205 (HXBC2 numbering). Deletions are retained relative to HXBC2 to distinguish insertions. Loop2b is shown in grey. **(C)** Summary table of SPR KD values for ApexGT variants and mature or reverted members of the PCT64 and PG9/PG16 class of antibodies. K_Ds were measured with trimer analytes and IgG ligands on the sensor chip and were determined by fitting the data with a 1:1 binding model. A dash indicates that the variant was not tested. A “>” or “>*” indicates that binding was not detected at maximal SOSIP concentration of 5 uM or 10 uM, respectively. All KD values are stated in nM. (related to **Fig 4**)

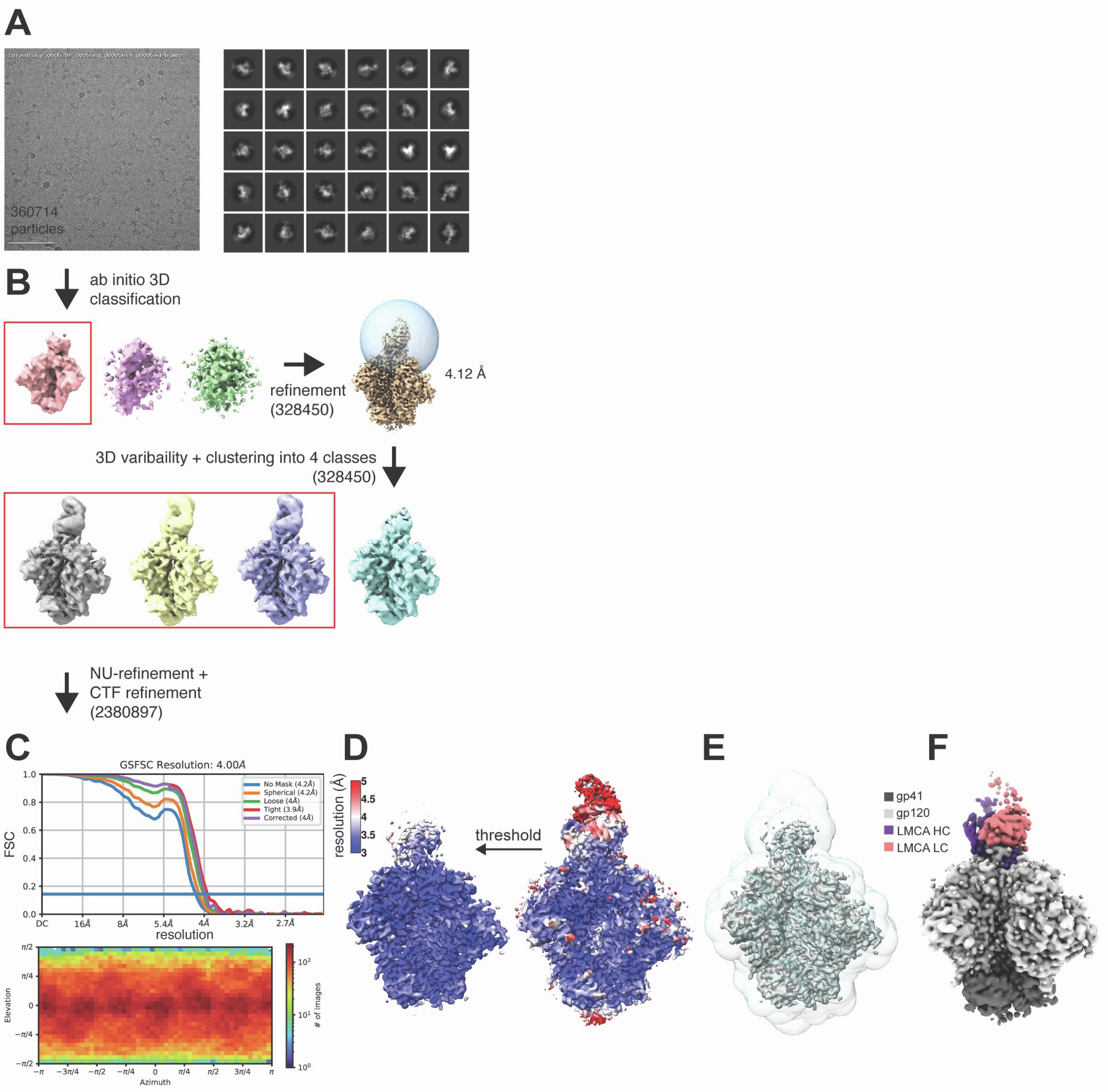

Figure S3. Cryo-EM data processing workflow for ApexGT2.2MUT in complex with PCT64 LMCA Fab illustrates the steps of structure determination.

(A) A representative raw micrograph and 2-D class averages of picked particles. (B) Flowchart of 3-D data processing steps. (C) Fourier Shell Correlation (FSC) curve and particle angular distribution plots, (D) local resolution estimates, (E) soft mask used during refinement and FSC calculations, and (F) segmentation of the final 3-D reconstruction (related to **Fig 5**)

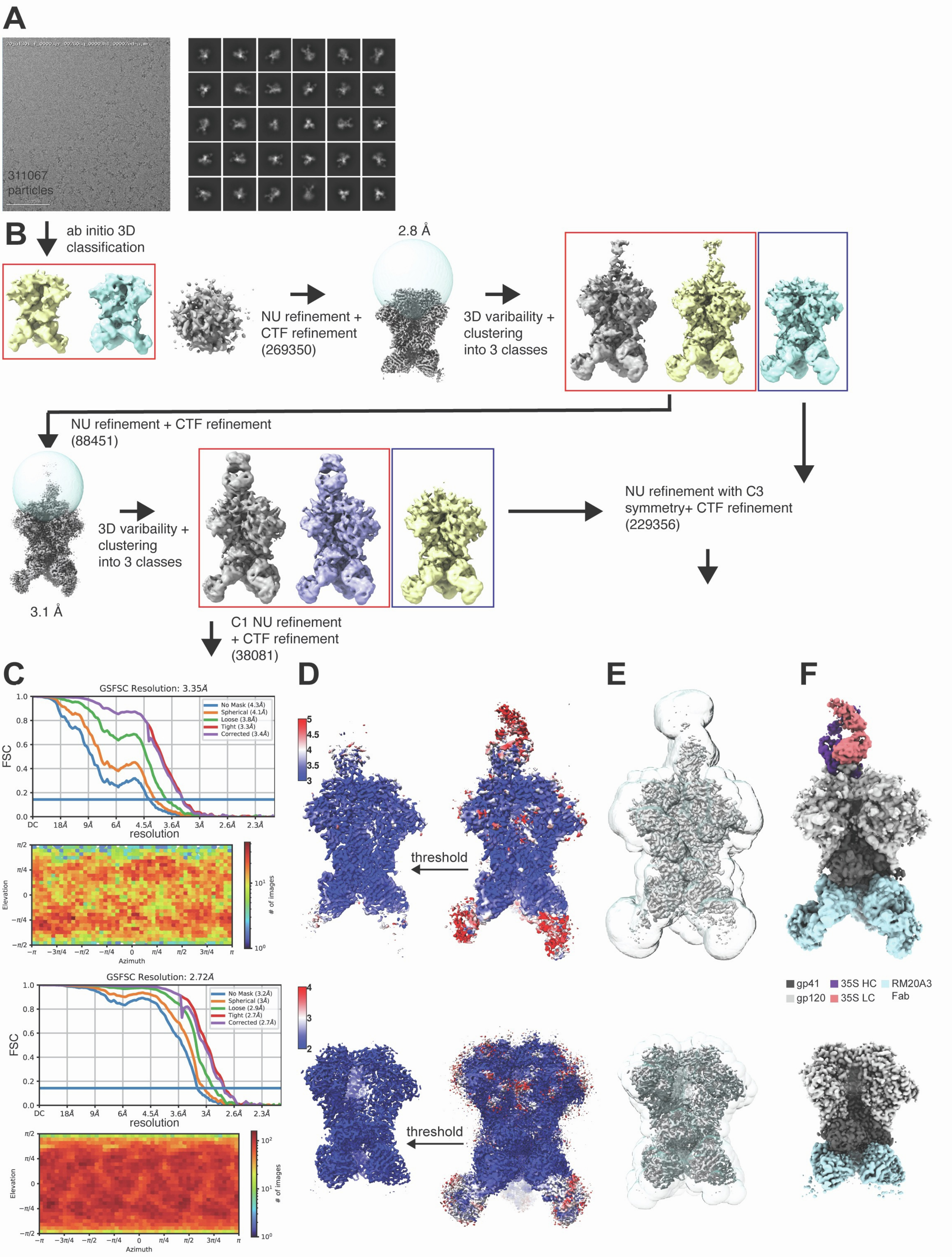

A
311067
particles
B
ab initio 3D
classification
NU refinement +
CTF refinement
(269350)
2.8 Å
3D varibaility +
clustering
into 3 classes
NU refinement + CTF refinement
(88451)
3D varibaility +
clustering
into 3 classes
3.1 Å
NU refinement with C3
symmetry+ CTF refinement
(229356)
C1 NU refinement
+ CTF refinement
(38081)
C
D
E
F
GSFSC Resolution: 3.35Å
No Mask (4.3Å)
Spherical (4.1Å)
Loose (3.8Å)
Tight (3.3Å)
Corrected (3.4Å)
FSC
resolution
Elevation
Azimuth
# of images
threshold
gp41
gp120
35S HC
35S LC
RM20A3
Fab
GSFSC Resolution: 2.72Å
No Mask (3.2Å)
Spherical (3Å)
Loose (2.9Å)
Tight (2.7Å)
Corrected (2.7Å)
FSC
resolution
Elevation
Azimuth
# of images
threshold

Figure S4. Cryo-EM data processing workflow for ApexGT2 in complex with PCT64 35S and RM20A3 Fabs illustrates structure determination assisted by base-directed Fab.

(A) A representative raw micrograph and 2-D class averages of picked particles. **(B)** Flowchart of 3-D data processing steps. **(C)** FSC curve and particle angular distribution plots, (D) local resolution estimates, (E) soft mask used during refinement and FSC calculations, and (F) segmentation of the final 3-D reconstructions both with and without 35S Fab. The base-binding antibody RM20A3 Fab, included in the PCT64 35S complex to improve angular distribution and resolution, is evident in (B)-(F) but for simplicity is not shown in Figure 5. (related to **Fig 5**)

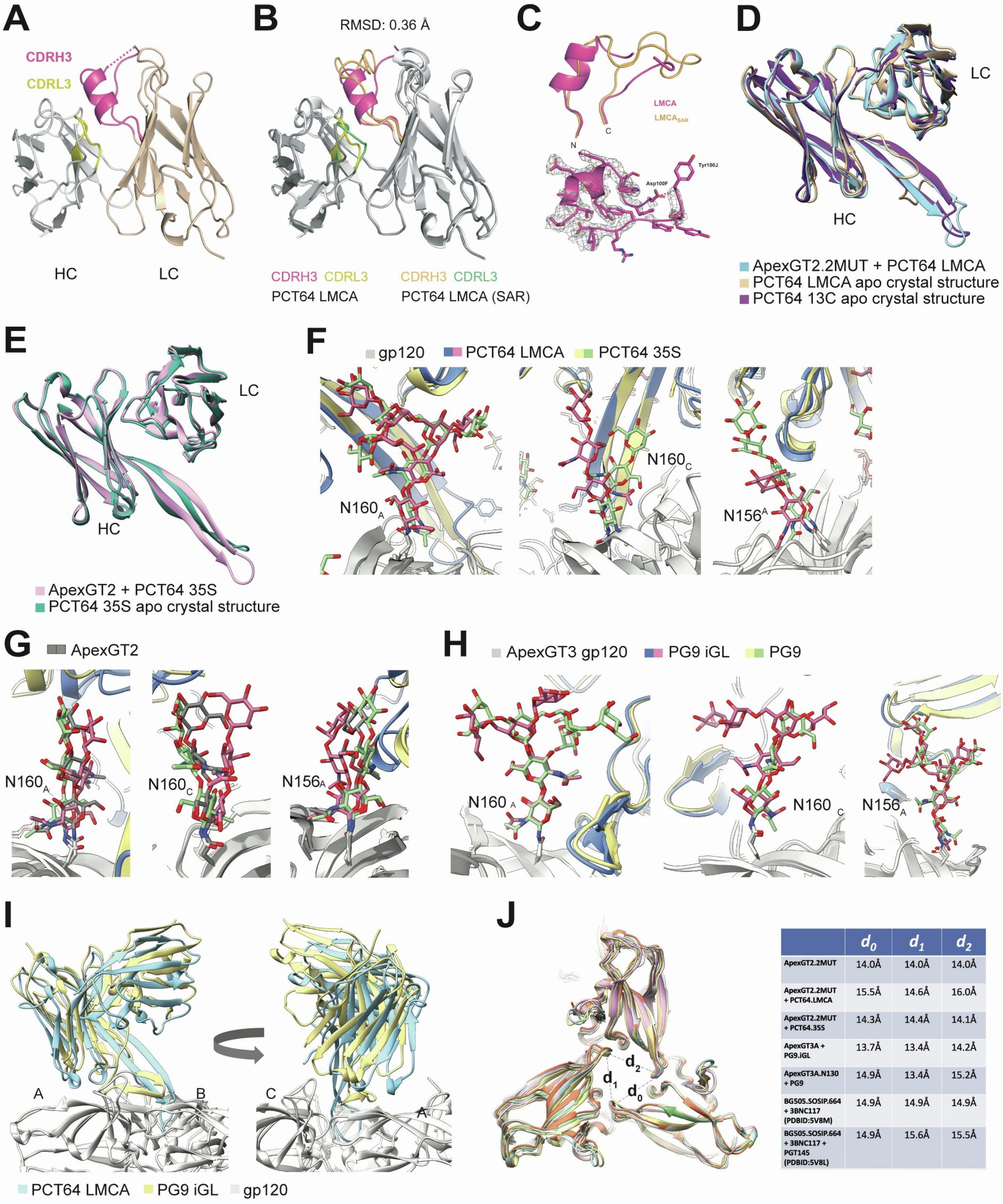

	d_0	d_1	d_2
ApexGT2.2MUT	14.0Å	14.0Å	14.0Å
ApexGT2.2MUT + PCT64.LMCA	15.5Å	14.6Å	16.0Å
ApexGT2.2MUT + PCT64.35S	14.3Å	14.4Å	14.1Å
ApexGT3A + PG9.iGL	13.7Å	13.4Å	14.2Å
ApexGT3A.N130 + PG9	14.9Å	13.4Å	15.2Å
BG505.SOSIP.664 + 3BNC117 (PDBID:5V8M)	14.9Å	14.9Å	14.9Å
BG505.SOSIP.664 + 3BNC117 + PGT145 (PDBID:5V8L)	14.9Å	15.6Å	15.5Å

Figure S5. Extended structural analysis provides detailed views of additional important structural features.
(A) Crystal structure of the PCT64 LMCA variable region. **(B)** Cα alignment of the LMCA (dark gray) and LMCASAR (white) variable light (VL) domains. The 'SAR' designation refers to the three mutations in the LCDR3. CDR loops of LMCA colored as in (A); HCDR3 of LMCASAR in orange, and LCDR3 in green. **(C)** Alignment of the HCDR3 in LMCA and LMCASAR upon superposition of the VH regions. The former HCDR3 is colored in magenta, and the latter in orange (top), and electron density map (bottom) of the LMCA HCDR3. Intervening residues between Asp100F and Tyr100J are missing electron density. 2Fo-Fc maps contoured at 1.0σ. **(D)** PCT64 LMCA structure from cryo-EM complex and the apo crystal structures of PCT64 LMCA, LMCASAR (PDBID:6CA9), and PCT64-13C (PDBID:6CA7) aligned to the heavy chains. **(E)** PCT64 35S structure from cryo-EM complex and the apo crystal structure of PCT64 35S (PDBID:6CA6) aligned to the heavy chains. **(F)** Both PCT64 cryo-EM structures aligned to the HC to highlight changes in glycan conformations. **(G)** PCT64 structures aligned to the ApexGT2 with unbound trimer apex structure (dark gray) showing the displacement of each apex glycan relative to its unbound trimer apex conformation. **(H)** Same as in (F) but for PG9 and PG9 iGL. **(I)** PG9 iGL and PCT64 LMCA structures aligned to gp120 showing their near identical angle of approach. **(J)** Interprotomer distances as measured between the alpha carbons of residue 167. (related to **Figs 5** and **6**)

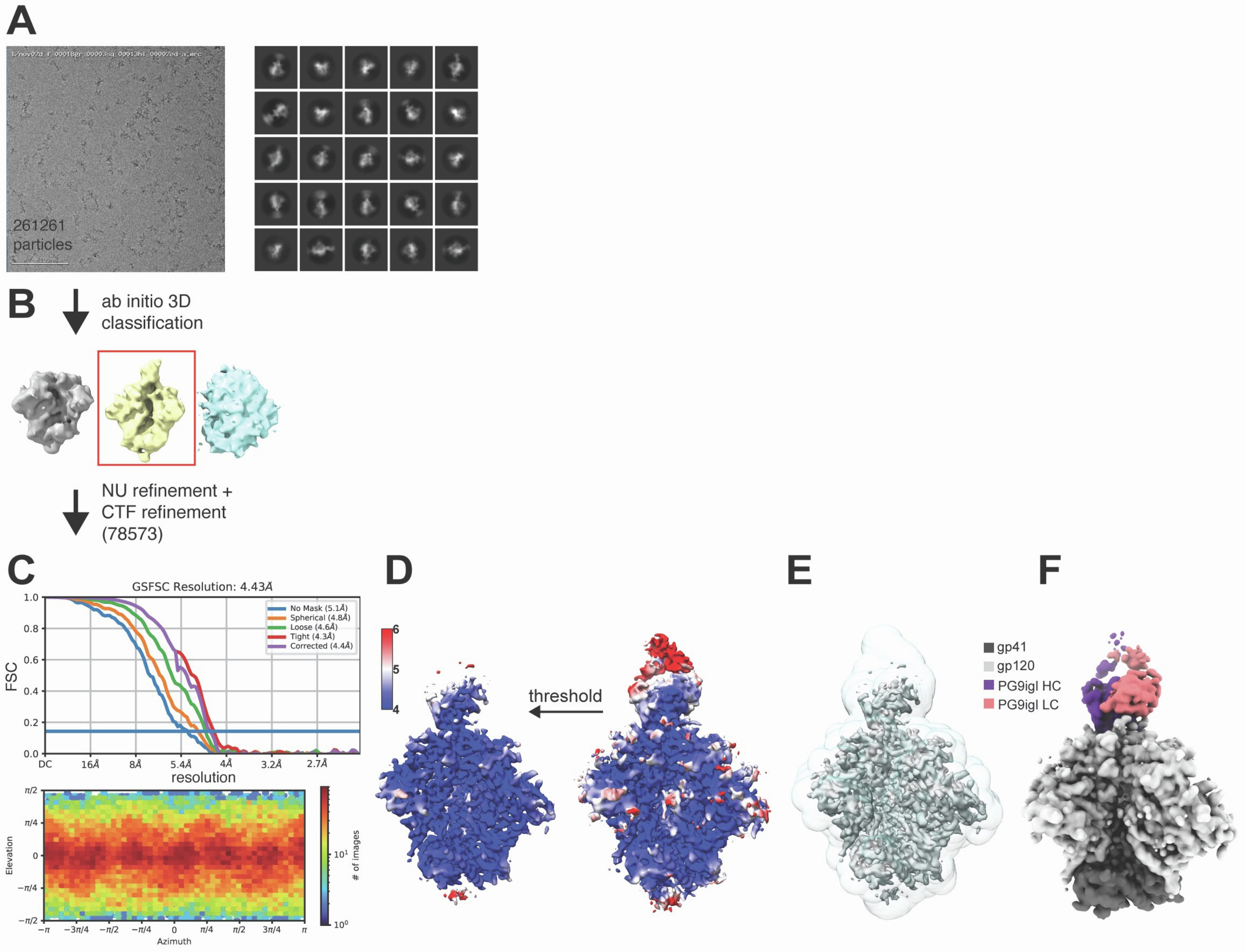

Figure S6: Cryo-EM data processing workflow for ApexGT3A in complex with PG9 iGL Fab illustrates steps of structure determination. **(A)** A representative raw micrograph and 2-D class averages of picked particles. **(B)** Flowchart of 3-D data processing steps. **(C)** FSC curve and particle angular distribution plots, **(D)** local resolution estimates, **(E)** soft mask used during refinement and FSC calculations, and **(F)** segmentation of the final 3-D reconstruction. (related to **Fig 6**)

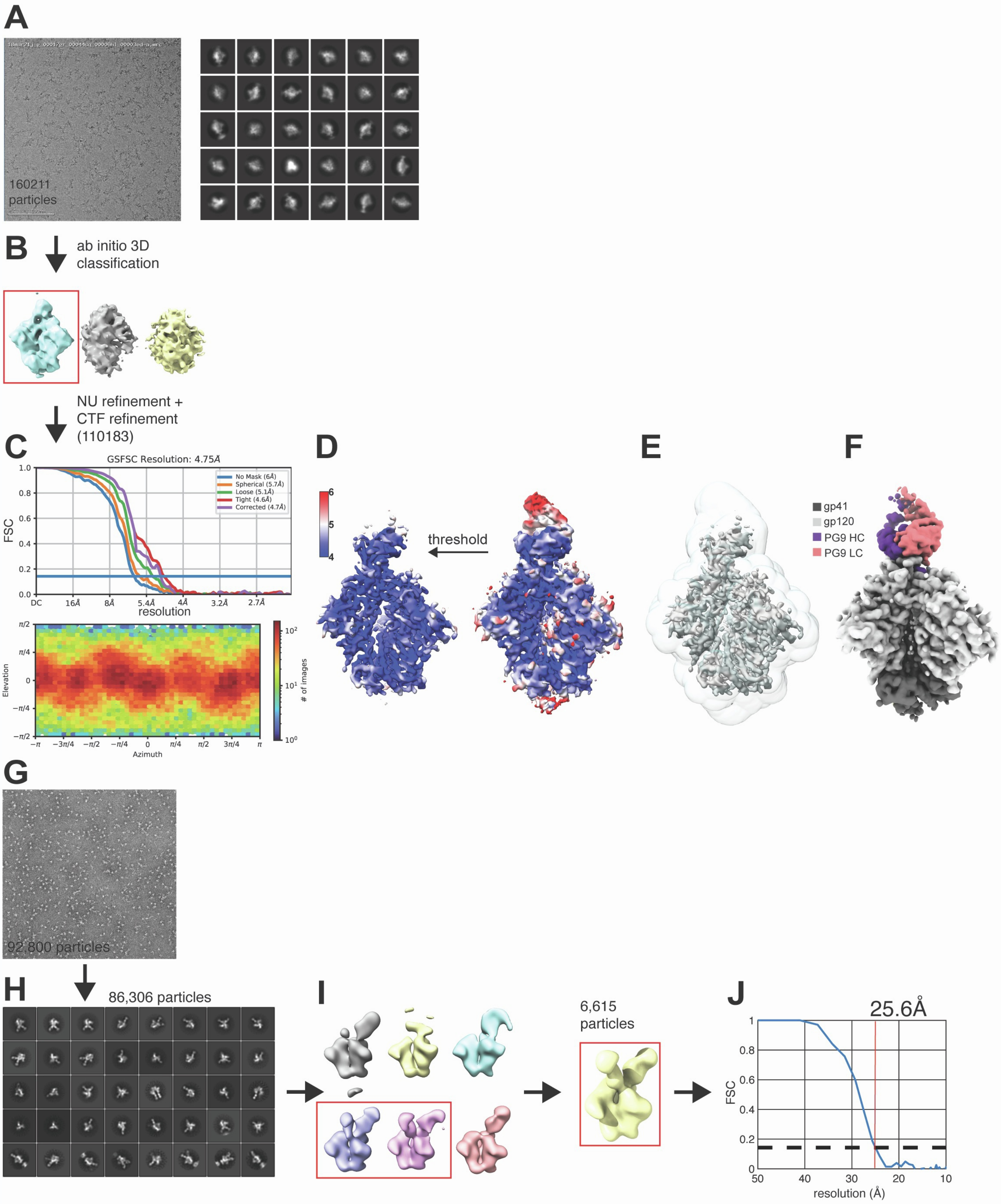
A
160211 particles
B
ab initio 3D classification
NU refinement + CTF refinement (110183)
C
GSFSC Resolution: 4.75Å
No Mask (6Å)
Spherical (5.7Å)
Loose (5.1Å)
Tight (4.6Å)
Corrected (4.7Å)
FSC
resolution
Elevation
Azimuth
# of images
D
threshold
E
F
gp41
gp120
PG9 HC
PG9 LC
G
92,800 particles
H
86,306 particles
I
6,615 particles
J
25.6Å
FSC
resolution (Å)

Figure S7. Cryo-EM data processing workflow for ApexGT3A.N130 in complex with PG9 Fab illustrates steps of structure determination.

(A) A representative raw micrograph and 2-D class averages of picked particles. **(B)** Flowchart of 3-D data processing steps. **(C)** FSC curve and particle angular distribution plots, **(D)** local resolution estimates, **(E)** soft mask used during refinement and FSC calculations, and **(F)** segmentation of the final 3-D reconstruction. G) Representative negative stain EM micrograph. H) negative stain 2-D class averages of ApexGT3 in complex with PG9 Fab. I) negative stain 3-D classification and refinement of 2-Fab bound class. J) Fourier shell correlation resolution plot for 2-Fab bound class. (related to **Fig 6**)

Series	mAB	HCDR3	Templated Amino Acids				Neutralization						
			HCDR3			VH Gene	Publication		CATNAP			Seaman	
			Templated (AA)	Template Ratio	Template Normal Ratio	Mutations (AA)	Breath (%)	Potency (mg/mL)	Breath (%)	Potency (mg/mL)	Viruses Tested	Breath (%)	Potency (mg/mL)
	VDJ	TT---YYDFWSGYY--YYYYYYMDV	**20**	**0.80**									
	iDG	..GVET........DH.........	19	0.76	0.95	0	0	-	-	-	-	-	-
	PCT64.LMCA	..GVET.......DDH..D..FR..	15	0.60	0.75	0	-	-	-	-	-	-	-
	PCT64.13A	..GVET.......DDH..D..F...	16	0.64	0.80	5	-	-	-	-	-	-	-
	PCT64.13B	.AGVET.......DDH..D......	16	0.64	0.80	8	-	-	-	-	-	-	-
	PCT64.13C	..GVET.......DDH..D..FK..	15	0.60	0.75	6	0	-	-	-	-	-	-
	PCT64.13D	..GVET.....A.RDD..D......	16	0.64	0.80	9	0	-	-	-	-	-	-
	PCT64.13E	M.GVETR.......DD..DH.F...	14	0.56	0.70	11	0	-	-	-	-	-	-
	PCT64.13F	M.GVET.....A.DDH..D..FT..	13	0.52	0.65	7	0	-	-	-	-	-	-
	PCT64.13G	M.GVETK.......DD..DH.FI..	13	0.52	0.65	11	0	-	-	-	-	-	-
	PCT64.13H	M.GVETR.......DD..DH.FI..	13	0.52	0.65	12	0	-	-	-	-	-	-
	PCT64.18A	M.GVET.S..HDDRDD..D......	12	0.48	0.60	14	0	-	-	-	-	-	-
	PCT64.18B	M.GVETK...GDD.GD..DH.FI..	10	0.40	0.50	16	14	0.36	-	-	-	-	-
	PCT64.18C	M.GVETG....DD.AN..DP.LI..	11	0.44	0.55	15	8	4.00	-	-	-	-	-
	PCT64.18D	M.GVETG....D..AQ..DP.LI..	12	0.48	0.60	17	22	0.23	-	-	-	23	0.52
	PCT64.18E	M.GVETG....DD.GN..DP.LI..	11	0.44	0.55	15	-	-	-	-	-	-	-
	PCT64.18F	M.GVETG....D..AQ..DP.LI..	12	0.48	0.60	17	22	0.37	-	-	-	-	-
	PCT64.24A	N.GVES.....DD.SQ.Q.K.FW..	12	0.48	0.60	17	19	2.90	-	-	-	-	-
	PCT64.24B	N.GVES.....DD.SQ.Q.K.FW..	12	0.48	0.60	17	16	4.00	-	-	-	-	-
	PCT64.24C	N.GVES.....DD.AQ.E.K.FR..	12	0.48	0.60	17	-	-	-	-	-	-	-
	PCT64.24D	N.GVET.....A.DDS..DK.FT..	12	0.48	0.60	16	-	-	-	-	-	-	-
	PCT64.24E	N.GVES.....DD.SQ.D.T.LS..	12	0.48	0.60	18	16	3.90	-	-	-	26	0.52
PCT64	PCT64.24F	M.GVETG....DE.SQH.DP.LI..	10	0.40	0.50	17	22	0.14	-	-	-	24	0.99
	PCT64.24G	M.GVETG....DD.SQH.NS.LI..	10	0.40	0.50	19	22	2.30	-	-	-	-	-
	PCT64.24H	M.GVETG....DD.SQH.NS.LI..	10	0.40	0.50	19	16	2.90	-	-	-	-	-
	PCT64.35A	M.GVETK.......DD..DH.FI..	13	0.52	0.65	11	0	-	-	-	-	-	-
	PCT64.35B	M.GVEKG....DD.SQH.NT.LI..	10	0.40	0.50	18	24	0.23	-	-	-	28	1.10
	PCT64.35C	M.GVEKG....DD.SQH.NT.LI..	10	0.40	0.50	18	24	0.51	-	-	-	-	-
	PCT64.35D	M.GVEKG....DD.SQH.NT.LI..	10	0.40	0.50	18	24	0.31	-	-	-	29	1.30
	PCT64.35E	M.GVEKG....DD.SQH.NT.LI..	10	0.40	0.50	18	24	0.29	-	-	-	-	-
	PCT64.35F	M.GVEKG....DD.SQH.NT.LI..	10	0.40	0.50	19	24	0.53	-	-	-	-	-
	PCT64.35G	M.GVEKG....DD.SQH.NT.LI..	10	0.40	0.50	19	24	0.44	-	-	-	28	2.10
	PCT64.35H	M.GVERG....DD.SQH.NT.LI..	10	0.40	0.50	19	19	1.30	-	-	-	-	-
	PCT64.35I	M.GVERGG...DE.SQHWDP.LI..	8	0.32	0.40	19	5	5.70	-	-	-	-	-
	PCT64.35K	M.GVERG....DD.SQH.NT.LI..	10	0.40	0.50	19	16	0.23	-	-	-	-	-
	PCT64.35J	M.GVEAG....DE.SQH.NT.LI..	10	0.40	0.50	21	-	-	-	-	-	-	-
	PCT64.35L	M.GVERG....DD.SQH.NT.LI..	10	0.40	0.50	19	-	-	-	-	-	-	-
	PCT64.35M	N.GVEW.....DE.SQEETK.FK..	10	0.40	0.50	19	32	0.46	-	-	-	26	0.41
	PCT64.35N	M.GVEAG....DE.SQH.NT.LI..	10	0.40	0.50	21	16	0.83	-	-	-	-	-
	PCT64.35O	M.GVEAG....DE.SQH.NT.LI..	10	0.40	0.50	21	19	0.06	-	-	-	23	1.40
	PCT64.35P	M.GVERG....DD.SQH.NT.LI..	10	0.40	0.50	19	-	-	-	-	-	-	-
	PCT64.35Q	M.GVEAG....DE.SQH.NT.LI..	10	0.40	0.50	21	-	-	-	-	-	-	-
	PCT64.35R	M.GVEKG....DD.SQH.NT.LI..	10	0.40	0.50	20	-	-	-	-	-	-	-
	PCT64.35S	M.GVERG....DD.SQH.NT.LI..	10	0.40	0.50	21	22	0.13	-	-	-	27	0.62
	PCT64.35T	M.GVERG....DD.SQH.NT.LI..	10	0.40	0.50	22	-	-	-	-	-	-	-
	VDJ	AR-----------YYYGSGSYWYFDL	**15**	**0.58**									
	CH01	..GTDYTIDDAGIH.Q...TF.....	11	0.42	0.73	28	45	0.75	52	1.13	242	-	-
CH	CH02	..GTDYTIDDQGRF.Q...TF....F	10	0.38	0.67	22	36	0.94	36	0.94	90	33	0.51
	CH03	..GTDYTIDDQGIF.K...TF.....	11	0.42	0.73	22	44	0.47	44	0.47	90	-	-
	CH04	..GTDYTIDDQGIR.Q...TF....V	10	0.38	0.67	23	44	0.74	44	0.92	117	10	0.38
	VDJ	AR------------YYDFWSGYYYYYYMDV	**18**	**0.60**									
PG9/PG16	PG9	V.EAGGPDYRNGYN....YD...N.H....	13	0.43	0.72	19	78	0.28	75	0.18	731	62	0.24
	PG16	..EAGGPIWHDDVK....ND...N.H....	14	0.47	0.78	21	74	0.19	73	0.11	565	53	0.04

Series	mAB	HCDR3	Templated Amino Acids				Neutralization						
			HCDR3			VH Gene	Publication		CATNAP			Seaman	
			Templated (AA)	Template Ratio	Template Normal Ratio	Mutations (AA)	Breath (%)	Potency (mg/mL)	Breath (%)	Potency (mg/mL)	Viruses Tested	Breath (%)	Potency (mg/mL)
	VDJ	AR----------DYGDY---------DYGDYMDV	**15**	**0.44**									
	PGT141	T.GSKHRLRDYVL.D..GLINYQEWN..LEFL..	8	0.24	0.53	27	55	0.33	55	0.30	186	-	-
	PGT142	T.GSKHRLRDYVL.D..GLINYQEWN..LEFL..	8	0.24	0.53	29	57	0.24	57	0.17	192	-	-
	PGT143	T.GSKHRLRDYVL.D..GLINYQEWN..LEFL..	8	0.24	0.53	27	56	0.34	55	0.25	264	-	-
	PGT144	TGGSKHRLRDYVL.D..GLINQQEWN..LEFL..	7	0.21	0.47	30	38	1.58	38	1.58	162	-	-
	PGT145	LTGSKHRLRDYFL.NE.G-PNYEEWG..LATL..	6	0.18	0.40	29	78	0.30	70	0.19	621	49	0.09
	PGDM1400	.KGSKHRLRDYAL.D.DGALNWAVDV..LSNLEF	5	0.15	0.33	45	83	0.01	77	0.03	396	60	0.02
	PGDM1401	..GSKHRLRDYVM.D..GALQWAVYV..LSNL..	9	0.26	0.60	42	63	0.01	63	0.01	107	-	-
PGT/PGDM	PGDM1402	.KGSKHRLRDYAL.D.IGALQWAVDV..LSTLEF	5	0.15	0.33	43	41	0.38	41	0.38	102	-	-
	PGDM1403	VKGSKFRLREWA..NEWG-LVSAQHG..VTQLGI	4	0.12	0.27	41	39	0.08	39	0.08	98	-	-
	PGDM1404	V.GAKFRLRHDAT.DYWNDLLWADDR..VTQL.L	5	0.15	0.33	40	83	0.09	35	0.09	88	-	-
	PGDM1405	V.GAKFRLRHDAT.DY.NDLLWADDR..VTQL.L	6	0.18	0.40	37	63	0.04	28	0.04	98	-	-
	PGDM1406	VKGQKFRLTEWA..NEFG-LVAAQKG..VTQL..	6	0.18	0.40	40	41	1.09	14	1.09	88	-	-
	PGDM1407	VKGQKFRLTEWA..NEFG-LVAAEKG..VTQL..	6	0.18	0.40	37	-	-	-	-	-	-	-
	PGDM1409	V.GAKFRLRHDAT.DYWNDLLWADDR..VTQL.L	5	0.15	0.33	37	-	-	-	-	-	-	-
	PGDM1410	V.GSKFRLRNDAI.DYWNDLLWADDG..VTKL.L	5	0.15	0.33	40	-	-	-	-	-	-	-
	PGDM1411	..RTEKQLRAEYVLDQED-GFYREEAI.ITVL..	5	0.15	0.33	31	-	-	-	-	-	-	-
	PGDM1412	VKGLKFRLREWS..NEFG-LVAAQHG..VTQ.E.	6	0.18	0.40	35	-	-	-	-	-	-	-
	VDJ	AR--------------YYDFWSGYY------------FDI	**14**	**0.35**									
	VRC26.UCA	.KDLGESENEEWAT-D....SI..PGQDPRG--VVGA...	10	0.25	0.71	0	0	0	-	-	-	-	-
	VRC26.01	.KDVGDYKSDEWGT-E...ISIS.PIQDPRA--MVGA..L	7	0.18	0.50	15	19	1.86	19	1.76	191	-	-
	VRC26.02	.KDIREYECEYWTS-D....GRPQPCIDSRG--VVGT..V	7	0.18	0.50	15	17	0.40	29	0.19	56	-	-
	VRC26.03	.KDLREDECEEWWS-D....GKQLPCRKSRG--VAGI..G	7	0.18	0.50	13	39	0.07	40	0.07	203	-	-
	VRC26.04	.KDLREDECEEWWS-D....GKQLPCRKSRG--VAGI..K	7	0.18	0.50	13	30	0.32	39	0.16	56	-	-
	VRC26.05	..DQRYYECEEWAS-D....GREQPCLDPRG--VVGI..L	8	0.20	0.57	19	21	0.10	32	0.09	56	-	-
	VRC26.06	..DLRELECEEWTLYN....G.RGPCVDPRG--VAGS..V	9	0.23	0.64	17	13	1.06	16	1.06	206	-	-
	VRC26.07	.KDLREDECEEWWS-D....GKKLPCRKSRG--VAGV..K	7	0.18	0.50	17	13	1.51	23	0.61	56	-	-
	VRC26.08	V.DQREDECEEWWS-D....GRELPCRKFRGLGLAGI...	8	0.20	0.57	15	47	0.03	58	0.02	403	28	0.01
	VRC26.09	VKDQREDECEEWWS-D....GRELPCRKSRGLGLAGI..M	6	0.15	0.43	20	47	0.07	50	0.03	153	31	0.01
	VRC26.10	.KDMREYECEYWTS-D....GRPQPCIDRRG--VVGI..M	7	0.18	0.50	16	23	0.60	34	0.26	56	-	-
	VRC26.11	VKDMRELECEEWAS-D....GKPQPCLDRRG--VSGISAW	4	0.10	0.29	21	26	0.94	36	0.38	56	-	-
	VRC26.12	..DLRESECEEWES-D....GKKGPCVKPRG--VAGGL.L	7	0.18	0.50	21	6	0.49	20	3.49	56	-	-
	VRC26.13	..DLRESECEEWES-D....GKKGPCVKPRG--VAGGL.L	7	0.18	0.50	20	8	0.13	17	2.98	53	-	-
	VRC26.14	..DVREMECEEWAS-D....GR.GPCRDFRG--VVGIL..	9	0.23	0.64	16	25	0.20	32	0.31	53	-	-
	VRC26.15	..DLREYECELWAS-D....GKPQPCEDPRG--VVGTS..	8	0.20	0.57	11	33	0.56	40	0.54	53	-	-
CAP256	VRC26.16	..DVREMECEEWAS-D....GR.GPCRDFRG--VVGIL..	9	0.23	0.64	17	29	0.37	36	0.38	53	-	-
	VRC26.17	..DMREMECEEWAS-D....GRPGPCRDLRG--VVGT...	9	0.23	0.64	18	29	0.13	36	0.15	53	-	-
	VRC26.18	..DMREMECEEWAS-D....GRSGPCRDFRG--VVGV...	9	0.23	0.64	18	27	0.33	34	0.42	53	-	-
	VRC26.19	..DSREYECELWTS-D....GKPQPCIDTRD--VGGL..M	8	0.20	0.57	18	46	0.24	51	0.26	53	-	-
	VRC26.20	V.DQREDECEERWS-D..H.GRVLPCRKYRGLGLAGV...	7	0.18	0.50	20	2	1.87	2	1.87	48	-	-
	VRC26.21	V.DRQEDECEEWWS-D..A.GR.GPCRKYHGQGLAGI...	8	0.20	0.57	27	15	0.14	23	0.12	53	-	-
	VRC26.22	V.DRQEDECVEWWS-D..N.GRELPCSKFRGLGLAGI...	7	0.18	0.50	23	46	0.06	51	0.06	53	-	-
	VRC26.23	.KDLREYECEEWRS-D....GRDQPCIDSQG--VVGIL..	7	0.18	0.50	17	8	0.10	17	1.25	53	-	-
	VRC26.24	.KDLGGIKNDEWGT-D...ISVS.PVQDPRA--VAGI..V	7	0.18	0.50	9	8	0.40	8	0.40	48	-	-
	VRC26.25	.KDLREDECEEWWS-D....GKQLPCAKSRG-GLVGIA.N	6	0.15	0.43	15	59	0.00	65	0.00	374	-	-
	VRC26.26	V.DQREDECEEWWS-D....GKELPCRKFRGLGLAGI..V	7	0.18	0.50	20	57	0.03	58	0.03	203	-	-
	VRC26.27	V.DQREDECEEWWS-D....GKELPCRKFRGLGLAGI...	8	0.20	0.57	19	54	0.03	55	0.01	203	-	-
	VRC26.28	V.DRQEDECVEWWS-D..N.GRELPCSKFRGLGLAGI...	7	0.18	0.50	21	48	0.01	53	0.05	53	-	-
	VRC26.29	V.DRQEDECVEWWS-D..N.GRELPCSKFRGLGLAGI...	7	0.18	0.50	21	50	0.05	55	0.10	53	-	-
	VRC26.30	V.DRQEDECEEWWS-D..N.GRELPCRKFRGQGLAGI...	7	0.18	0.50	21	35	0.08	42	0.10	53	-	-
	VRC26.31	V.DRQEDECEEWWS-D..N.GRELPCRKFRGPGLAGI...	7	0.18	0.50	20	23	0.15	30	0.09	53	-	-
	VRC26.32	VKDLRELECEEWTS-D....GRPQPCIDPRG--VSGISAM	4	0.10	0.29	18	21	0.12	28	0.22	53	-	-
	VRC26.33	..DQRYYECEEWAS-D....GREQPCQDPRG--VVGI...	9	0.23	0.64	17	23	0.12	30	0.24	53	-	-

Table S1: V2-Apex bnAb HCDR3s and neutralization.

Comprehensive V2-apex HCDR3 alignments and neutralization details for all V2 mABs considered in this study. The VDJ junction is shown for each bnAb class from Figure 2A. Dashes indicate a junction that cannot be assigned to a gene segment. HCDR3 template metrics are shown next to each mAb. The templated portion indicates how many amino acids match the VDJ junction. Templated ratio is the number of templated amino acids divided by the total length of the HCDR3. VH gene mutations are shown in number of amino acids mutated from the parent VH gene. Neutralization metrics are shown from three different sources. The publication column indicates the source of neutralization breath and potency. CATNAP is taken from the CATNAP webservice which is a compiled list of publications where that antibody was tested (Yoon et al., 2015). Data from the Seaman 109- virus panel are shown for many V2 -apex members (Landais et al., 2017). (related to **Fig 2**)

Series	HCDR3 Length (AA)	$D_{start + Match}$	V_{Fam}	J_{Gene}	V_k/V_l	V_{gene*}	D_{gene*}
**PCT64**	25	......[YRKG][DSG]FWS..............	3	J6	K3-20	3-15	3-3
**CH01-CH04**	26	..............Y[YQK]GSG.......	3	J2	K3-20	3-20	3-10
**PG9/16**	30	...............YDF............	3	J6	L2-14	3-33	3-3
**PGT/PGDM**	33	.............Y[GND][DEY].................	1	J6	K2-28	1-8	4-17
**PGT/PGDM**	34	.............Y[GND][DEY]..................					
**CAP256**	**37**	................YD[FIL]..................	3	J3	L1-51	3-30	3-3
**CAP256**	**38**	.................YD[FIL]..................					
**CAP256**	**39**	................YD[FIL]....................					

Table S2: Precursor frequency query definitions.
The query precursor definitions for the each V2-apex series are shown. The HCDR3 lenghts are the lengths considered for that class. The D start and match are the regular expression syntax that was used to interrogate our database (see STAR methods). "." indicates a wildcard character used in the database search and will return all 20 amino acids at that position. The amino acids in between the brackets indicate the allowed mutations at that position (e.g. [DSG] will return an aspartic acid (D), serine (S) or glycine (G) at that position). V-gene and D- gene are shown for clarity but were not used in the query definition. VK/VL were used to interrogate pairing frequency for a given V gene family to a light chain from Dekosky et al. (DeKosky et al., 2015) (related to **Figs 2** and **3**)

Class	Donor	Match				
		Length	Length + D	Length + D + V	Length + D+V+J	Length + D+V+J (L)
PCT64	1	2900.780	48.960	39.410	28.560	5.526
	2	9790.950	186.660	67.340	55.430	10.725
	3	6533.440	428.330	256.260	216.760	41.944
	4	5708.780	100.940	31.190	21.550	4.171
	5	3698.460	31.440	12.970	11.990	2.319
	6	6104.710	65.270	31.200	28.670	5.548
	7	5635.160	48.010	12.360	7.880	1.525
	8	4325.100	80.810	23.140	16.070	3.110
	9	5265.640	160.780	67.970	56.450	10.924
	10	7656.000	219.190	86.210	62.380	12.071
	11	2636.440	18.090	8.900	7.120	1.378
	12	6652.290	86.580	23.140	19.670	3.807
	13	4092.680	50.370	21.000	14.500	2.805
	14	7336.020	71.740	22.750	17.240	3.337
	Median	5,671.968	76.275	27.165	20.614	3.989
	Geometric Mean	5,258.890	82.050	32.410	25.110	4.860
CH01-CH04	1	1659.520	0.640	0.550	-	-
	2	6208.890	2.150	0.430	-	-
	3	3972.970	1.820	1.630	-	-
	4	3276.010	0.720	0.070	-	-
	5	1888.630	0.200	-	-	-
	6	3639.380	2.870	1.010	0.170	0.033
	7	3508.070	7.340	2.690	-	-
	8	2621.550	8.550	0.800	-	-
	9	3603.590	1.230	0.330	-	-
	10	4747.040	2.200	0.280	-	-
	11	1640.240	0.950	0.170	-	-
	12	4534.700	2.830	0.570	-	-
	13	2294.700	2.350	0.880	0.020	0.005
	14	3935.610	2.620	0.860	-	-
	Median	3,555.829	2.177	0.559	0.000	0.000
	Geometric Mean	3,156.358	1.764	0.548	0.064	0.012
PG9/PG16	1	169.880	0.400	0.370	0.130	0.006
	2	757.990	2.060	0.750	0.370	0.018
	3	411.700	2.200	1.290	0.150	0.007
	4	406.360	2.260	0.860	0.230	0.011
	5	479.480	0.200	-	-	-
	6	740.090	1.860	1.350	0.510	0.025
	7	591.690	1.070	0.180	-	-
	8	475.870	0.910	-	-	-
	9	452.520	1.880	0.900	0.490	0.024
	10	616.170	1.330	0.600	0.140	0.007
	11	286.900	0.170	0.060	-	-
	12	1380.940	1.810	0.340	0.340	0.017
	13	235.630	0.850	0.330	0.060	0.003
	14	391.910	0.930	0.200	-	-
	Median	464.195	1.203	0.356	0.135	0.007
	Geometric Mean	464.790	0.989	0.438	0.220	0.011
PGT/PGDM	1	84.470	0.060	0.010	0.010	0.000
	2	247.050	0.880	0.480	0.400	0.015
	3	151.560	0.070	0.050	0.050	0.002
	4	154.950	0.670	0.390	0.390	0.015
	5	424.650	-	-	-	-
	6	535.500	-	-	-	-
	7	357.380	0.540	-	-	-
	8	335.790	0.110	-	-	-
	9	187.170	0.820	-	-	-
	10	247.880	0.920	0.500	-	-
	11	196.510	1.390	-	-	-
	12	350.530	0.980	0.300	0.190	0.007
	13	88.310	0.040	0.010	-	-
	14	160.550	0.400	0.190	0.140	0.006
	Median	221.782	0.469	0.012	0.000	0.000
	Geometric Mean	219.587	0.338	0.118	0.115	0.004
CAP256	1	47.240	-	-	-	-
	2	51.330	0.360	0.080	-	-
	3	73.920	0.780	0.690	-	-
	4	63.150	0.070	0.010	-	-
	5	277.270	0.200	-	-	-
	6	308.480	0.170	-	-	-
	7	165.170	-	-	-	-
	8	207.330	0.570	0.460	-	-
	9	58.410	0.160	0.160	-	-
	10	75.740	-	-	-	-
	11	91.880	0.060	-	-	-
	12	159.170	0.110	0.080	-	0.000
	13	25.040	0.050	0.040	0.020	0.003
	14	52.420	0.070	0.070	-	-
	Median	74.831	0.092	0.022	0.000	0.000
	Geometric Mean	91.346	0.156	0.092	0.025	0.003

Table S3: Individual donor precursor frequencies.

Individual precursor frequencies for each V2- apex bnAb class across donors, shown in counts per million. The columns deconstruct the precursor query by metric. Length, the HCDR3 length searched. Length+D, the HCDR3 length and D gene location described in Table S2. Length+D+V, the HCDR3 length, D gene location, and V gene family. Length+D+V+J (H), the HCDR3 length, D gene location, V gene family and J gene. H+L, the frequency computed from Length+D+V+J (H) and accounting for light chain pairing as described in the text. The median and geometric mean is shown for each V2-apex bnAb class. A dash indicates no results were found for that precursor definition. (related to **Figs 2** and **3**)

Class	Donor	Match			
		Length	Length + D	Length + D + V	Length + D+V+J
	**1**	20.2	14.9	14.9	14.2
	**2**	20.4	14.8	14.6	14.1
	**3**	19.5	14.4	14.4	14.0
	**4**	20.6	15.1	15.0	14.1
	**5**	20.4	14.3	13.9	13.7
	**6**	20.0	14.7	14.6	14.4
**PCT64**	**7**	20.5	15.3	15.4	14.8
	**8**	20.3	15.1	15.3	14.5
	**9**	19.9	14.7	14.6	14.2
	**10**	20.0	14.9	14.9	14.2
	**11**	20.4	14.6	14.5	14.0
	**12**	19.9	14.5	14.6	14.2
	**13**	20.3	15.2	15.2	14.4
	**14**	19.9	14.9	14.7	14.1
**Average**		20.2	14.8	14.8	14.2
	**1**	21.2	17.8	17.8	
	**2**	21.0	17.2	17.0	
	**3**	21.0	17.1	17.2	
	**4**	20.9	17.4	17.8	
	**5**	21.1	17.0		
	**6**	21.4	16.9	17.0	15.0
**CH01-CH04**	**7**	20.9	18.6	18.3	
	**8**	20.6	17.6	17.4	
	**9**	20.8	16.9	17.3	
	**10**	20.8	17.9	18.5	
	**11**	21.2	17.0	17.7	
	**12**	20.7	16.9	17.3	
	**13**	20.7	17.6	18.1	16.0
	**14**	20.2	17.5	17.7	
**Average**		20.9	17.4	17.6	15.5
	**1**	25.5	19.5	19.6	17.5
	**2**	24.1	19.7	19.8	19.1
	**3**	23.7	20.2	21.3	19.0
	**4**	24.1	19.7	19.7	18.9
	**5**	26.3	24.0		
	**6**	25.5	21.2	21.3	17.7
**PG9/PG16**	**7**	25.1	19.5	18.0	
	**8**	25.5	20.3		
	**9**	23.7	18.9	17.7	17.5
	**10**	23.8	19.7	19.7	19.0
	**11**	25.4	19.3	19.0	
	**12**	23.2	19.2	18.1	18.1
	**13**	24.0	20.6	20.1	18.6
	**14**	23.4	20.0	21.4	
**Average**		24.5	20.1	19.7	18.4
	**1**	29.9	27.2	26.0	26.0
	**2**	28.4	26.7	26.8	26.5
	**3**	29.1	27.0	27.3	27.0
	**4**	28.8	26.5	26.1	26.1
	**5**	30.0			
	**6**	29.7			
**PGT/PGDM**	**7**	29.2	26.0		
	**8**	29.9	28.0		
	**9**	28.6	27.3		
	**10**	28.5	27.3	27.0	
	**11**	29.4	26.5		
	**12**	28.8	25.5	26.6	25.0
	**13**	28.6	25.7	24.0	
	**14**	28.6	25.9	25.7	25.5
**Average**		29.1	26.6	26.2	26.0
	**1**	34.7			
	**2**	33.0	28.4	28.9	
	**3**	34.6	29.3	29.0	
	**4**	33.8	30.0	29.0	
	**5**	34.7	33.0		
	**6**	34.5	31.0		
**CAP256**	**7**	34.3			
	**8**	34.4	30.2	29.0	
	**9**	33.8	27.0	27.0	
	**10**	33.3			
	**11**	34.1	28.0		
	**12**	34.3	28.7	29.0	
	**13**	33.7	29.5	29.3	30.0
	**14**	33.8	30.2	30.2	
**Average**		34.1	29.6	28.9	30.0

Table S4: Individual donor precursor edit distances.

Individual mean edit distances for the precursors found in each donor from Table S3. An edit distance is the number of amino acid differences between a known bnAb from that class and the precursor found in our dataset. The mean edit distance is the average edit distance for all precursors found, given the precursor frequency definition in that column. The column definitions are described in Table S3. (related to **Figs 2** and **3**)

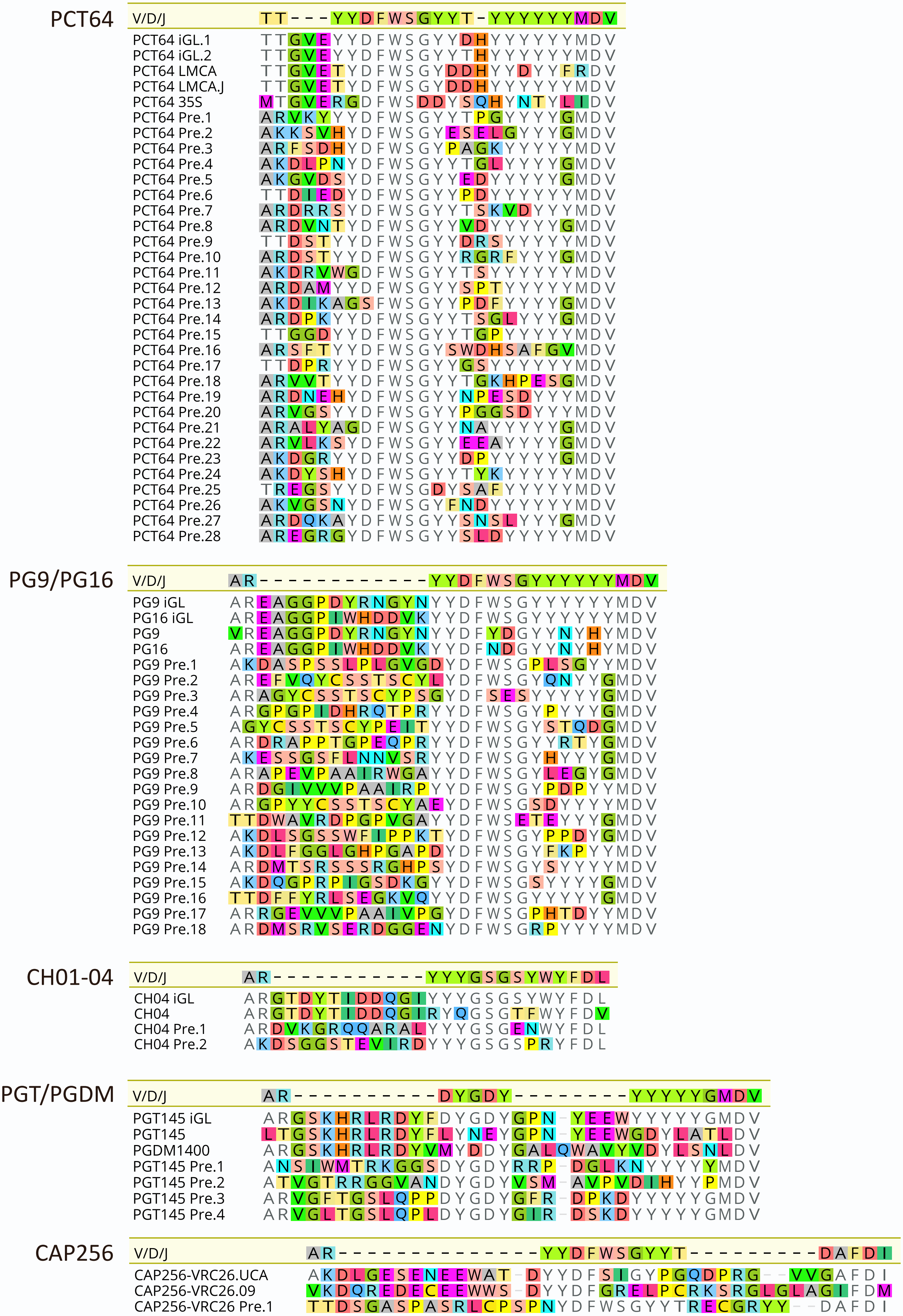

PCT64
V/D/J TT---YYDFWSGYYT-YYYYYYMDV
PCT64 iGL.1 TTGVEYYDFWSGYYDHYYYYYYMDV
PCT64 iGL.2 TTGVEYYDFWSGYYTHYYYYYYMDV
PCT64 LMCA TTGVETYDFWSGYDDHYYDYYFRDV
PCT64 LMCA.J TTGVETYDFWSGYDDHYYYYYYMDV
PCT64 35S MTGVERGDFWSDDYSQHYNTYLIDV
PCT64 Pre.1 ARVKYYYDFWSGYYTPGYYYYGMDV
PCT64 Pre.2 AKKSVHYDFWSGYESELGYYYGMDV
PCT64 Pre.3 ARFSDHYDFWSGYPAGKYYYYYMDV
PCT64 Pre.4 AKDLPNYDFWSGYYTGLYYYYGMDV
PCT64 Pre.5 AKGVDSYDFWSGYYEDYYYYYGMDV
PCT64 Pre.6 TTDIEDYDFWSGYYPDYYYYYYMDV
PCT64 Pre.7 ARDRRSYDFWSGYYTSKVDYYYMDV
PCT64 Pre.8 ARDVNTYDFWSGYYVDYYYYYGMDV
PCT64 Pre.9 TTDSTYYDFWSGYYDRSYYYYYMDV
PCT64 Pre.10 ARDSTYYDFWSGYYRGRFYYYGMDV
PCT64 Pre.11 AKDRVWGDFWSGYYTSYYYYYYMDV
PCT64 Pre.12 ARDAMYYDFWSGYYSPTYYYYYMDV
PCT64 Pre.13 AKDIKAGSFWSGYYPDFYYYYGMDV
PCT64 Pre.14 ARDPKYYDFWSGYYTSGLYYYGMDV
PCT64 Pre.15 TTGGDYYDFWSGYYTGPYYYYYMDV
PCT64 Pre.16 ARSFTYYDFWSGYSWDHSAFGVMDV
PCT64 Pre.17 TTDPRYYDFWSGYYGSYYYYYYMDV
PCT64 Pre.18 ARVVTYYDFWSGYYTGKHPESGMDV
PCT64 Pre.19 ARDNEHYDFWSGYYNPESDYYYMDV
PCT64 Pre.20 ARVGSYYDFWSGYYPGGSDYYYMDV
PCT64 Pre.21 ARALYAGDFWSGYYNAYYYYYGMDV
PCT64 Pre.22 ARVLKSYDFWSGYYEEAYYYYGMDV
PCT64 Pre.23 AKDGRYYDFWSGYYDPYYYYYGMDV
PCT64 Pre.24 AKDYSHYDFWSGYYTYKYYYYYMDV
PCT64 Pre.25 TREGSYYDFWSGDYSAFYYYYYMDV
PCT64 Pre.26 AKVGSNYDFWSGYFNDYYYYYYMDV
PCT64 Pre.27 ARDQKAYDFWSGYYSNSLYYYGMDV
PCT64 Pre.28 AREGRGYDFWSGYYSLDYYYYYMDV
PG9/PG16
V/D/J AR------------YYDFWSGYYYYYYMDV
PG9 iGL AREAGGPDYRNGYNYYDFWSGYYYYYYMDV
PG16 iGL AREAGGPIWHDDVKYYDFWSGYYYYYYMDV
PG9 VREAGGPDYRNGYNYYDFYDGYYNYHYMDV
PG16 AREAGGPIWHDDVKYYDFNDGYYNYHYMDV
PG9 Pre.1 AKDASPSSLPLGVGDYDFWSGPLSGYYMDV
PG9 Pre.2 AREFVQYCSSTSCYLYDFWSGYQNYYGMDV
PG9 Pre.3 ARAGYCSSTSCYPSGYDFSESYYYYYGMDV
PG9 Pre.4 ARGPGPIDHRQTPRYYDFWSGYPYYYGMDV
PG9 Pre.5 AGYCSSTSCYPEITYYDFWSGYSTQDGMDV
PG9 Pre.6 ARDRAPPTGPEQPRYYDFWSGYYRTYGMDV
PG9 Pre.7 AKESSGSFLNNVSRYYDFWSGYHYYYGMDV
PG9 Pre.8 ARAPEVPAAIRWGAYYDFWSGYLEGYGMDV
PG9 Pre.9 ARDGIVVVPAAIRPYYDFWSGYPDPYYMDV
PG9 Pre.10 ARGPYYCSSTSCYAEYDFWSGSDYYYYMDV
PG9 Pre.11 TTDWAVRDPGPVGAYYDFWSETEYYYGMDV
PG9 Pre.12 AKDLSGSSWFIPPKTYDFWSGYPPDYGMDV
PG9 Pre.13 AKDLFGGLGHPGAPDYDFWSGYFKPYYMDV
PG9 Pre.14 ARDMTSRSSSRGHPSYDFWSGYSYYYYMDV
PG9 Pre.15 AKDQGPRPIGSDKGYYDFWSGSYYYYGMDV
PG9 Pre.16 TTDFFYRLSEGKVQYYDFWSGYYYYYGMDV
PG9 Pre.17 ARRGEVVVPAAIVPGYDFWSGPHTDYYMDV
PG9 Pre.18 ARDMSRVSERDGGENYDFWSGRPYYYYMDV
CH01-04
V/D/J AR-----------YYYGSGSYWYFDL
CH04 iGL ARGTDYTIDDQGIYYYGSGSYWYFDL
CH04 ARGTDYTIDDQGIRYQGSGTFWYFDV
CH04 Pre.1 ARDVKGRQQARALYYYGSGENWYFDL
CH04 Pre.2 AKDSGGSTEVIRDYYYGSGSPRYFDL
PGT/PGDM
V/D/J AR----------DYGDY--------YYYYYGMDV
PGT145 iGL ARGSKHRLRDYFDYGDYGPN-YEEWYYYYYGMDV
PGT145 LTGSKHRLRDYFLYNEYGPN-YEEWGDYLATLDV
PGDM1400 ARGSKHRLRDYVMYDDYGALQWAVYVDYLSNLDV
PGT145 Pre.1 ANSIWMTRKGGSDYGDYRRP-DGLKNYYYYYMDV
PGT145 Pre.2 ATVGTRRGGVANDYGDYVSM-AVPVDIHYYPMDV
PGT145 Pre.3 ARVGFTGSLQPPDYGDYGFR-DPKDYYYYYGMDV
PGT145 Pre.4 ARVGLTGSLQPLDYGDYGIR-DSKDYYYYYGMDV
CAP256
V/D/J AR--------------YYDFWSGYYT---------DAFDI
CAP256-VRC26.UCA AKDLGESENEEWAT-DYYDFSIGYPGQDPRG--VVGAFDI
CAP256-VRC26.09 VKDQREDECEEWWS-DYYDFGRELPCRKSRGLGLAGIFDM
CAP256-VRC26 Pre.1 TTDSGASPASRLCPSPNYDFWSGYYTRECGRYY--DAFDI

Table S5. Alignment of example precursors identified by sequence database searching.
HCDR3 amino acid sequence alignment of identified precursors. Inferred germline, mature bnAb and corresponding V/D/J genes are shown for reference. Sequences shown are sampled from the full precursor pool to show diverse junctional residues. (related to **Figs 2** and **3**)

	ApexGT2.2MUT + PCT64 LMCA Fab	ApexGT2 + PCT64.35S Fab+ RM20A3 Fab	ApexGT2 + RM20A3 Fab	ApexGT3 + PG9 iGL Fab	ApexGT3.2MUT + PG9 Fab
	(EMDB-25732) (PDB ID 7T73)	(EMDB-25733) (PDB ID 7T74)	(EMDB-25734) (PDB ID 7T75)	(EMDB-25735) (PDB ID 7T76)	(EMDB-25736) (PDB ID 7T77)
Data Collection and Processing					
Electron microscope	Talos Arctica	Titan Krios	Titan Krios	Talos Arctica	Talos Arctica
Electron detector	K2 Summit	K2 Summit	K2 Summit	K2 Summit	K2 Summit
Magnification	36,000	29,000	29,000	36,000	36,000
Voltage (kV)	200	300	300	200	200
Electron exposure (e–/Å^2)	50	50	50	50	50
Defocus range (μm)	0.8 -3.0	0.5 -2.0	0.5 -2.0	0.8 -3.0	0.8 -3.0
Pixel Size (Å)	1.15	1.03	1.03	1.15	1.15
Symmetry imposed	C1	C1	C3	C1	C1
Initial particle images (no.)	360,271	311,067	311,067	261,261	160,211
Final particle images (no.)	238,078	38,081	229,656	78,573	110,183
Map resolution (Å)	4	3.35	2.72	4.43	4.75
FSC threshold	0.143	0.143	0.143	0.143	0.143
Map sharpening B factor (Å^2)	204.1	68.9	94.5	229.9	301.9
Model building and refinement					
Initial models used	(PDB ID: C6A9, Homology model from PDB ID: 6DFG)	(PDB ID: C6A6, PDB ID: 6X9S, ApexGT2.2MUT)	(PDB ID: C6A6, ApexGT2.2MUT)	(homology model from PDB ID: 3U4E, ApexGT2.2MUT)	(PDB ID: 3U4E, ApexGT3)
**Model composition**					
Protein Chains	8	14	4	8	8
Protein Residues	1907	2552	772	1934	1956
Ligands	78	98	29	104	105
**rms deviations**					
Bond Lengths (Å)	0.02	0.022	0.021	0.021	0.021
Bond angles (°)	1.872	1.729	1.784	1.923	1.762
**Ramachandran plot**					
Favored (%)	95.72	96.96	96.69	96.36	97.03
Disallowed (%)	0.8	0.52	0.79	0.26	0.26
**Validation**					
MolProbity score	1.44	0.9	0.96	1.08	10.96
Clashscore	2.22	0.67	0.79	1.23	0.97
Poor rotamers (%)	1.86	0.44	0.9	0.77	0.87
EMRinger score	1.95	3.52	4.46	1.11	0.66
Map-model cross correlation	0.79	0.86	0.82	0.8	0.79
CaBLAM outliers (%)	0.79	2.08	2.03	2.36	2.6

Table S6. Cryo-EM data and refined atomic statistics. Data collection and processing statistics for all cryo-EM and NS-EM datasets along with refinement and validation statistics for all atomic models. (related to **Figs 5** and **6**)
